# The potential effects, mechanisms and translational perspectives of natural products in the treatment of atopic dermatitis

**DOI:** 10.1186/s13020-026-01497-8

**Published:** 2026-09-16

**Authors:** Changyu Shao, Yuhao Zhang, Yining Bai, Yan Wu, Lin Cong, Mingming Zhao, Tianyang Ren

**Affiliations:** 1 Department of Pharmacy, Shengjing Hospital of China Medical University, No. 36, Sanhao Street, Shenyang, 110004 China; 2 Department of Dermatology, Shengjing Hospital of China Medical University, Shenyang, China; 3 Department of Pharmacy, The Second Affiliated Hospital of Shenyang Medical College, Shenyang, 110002 China

**Keywords:** Natural products, Atopic dermatitis, Inflammation, Permeability barrier, Pruritus

## Abstract

**Background:**

Atopic dermatitis (AD) is a common chronic inflammatory skin disorder with a continuously increasing global incidence. Current therapeutic options are often associated with adverse effects. In recent years, natural products have attracted considerable attention due to their anti-inflammatory and antioxidant properties, which can effectively alleviate the symptoms of AD.

**Objective:**

This study provides a comprehensive review of the research progress on the use of natural ingredients for the treatment of AD both domestically and internationally, with an emphasis on their efficacy and advantages in the context of AD pathogenesis. The aim is to offer a reference for the development of herbal ingredients as safer complementary or alternative therapeutic agents for AD.

**Methods:**

A comprehensive literature search was performed using key terms such as “atopic dermatitis”, “natural products”, “anti-inflammatory”, “skin barrier dysfunction”, “anti-pruritic”, “antimicrobial”, “antioxidant”, “gut microbiota”, and their combinations in databases including CNKI, PubMed, and Google Scholar. We systematically evaluated the pathogenesis and therapeutic mechanisms of AD, with a particular emphasis on in vivo studies.

**Results:**

Research has demonstrated that natural ingredients exhibit multifaceted advantages in the treatment of AD, including anti-inflammatory effects, skin barrier repair, anti-pruritic activity, modulation of the skin microbiota, alleviation of oxidative stress, and regulation of gut microbiota.

**Conclusion:**

We discuss and analyze various bioactive natural ingredients capable of modulating cytokines and signaling pathways, while also identifying their major translational limitations, such as standardization and safety issues. This review provides a mechanistic and evidence-based reference for developing natural products as safe, adjunctive therapies for AD.

## Introduction

Atopic Dermatitis (AD) is a prevalent, chronic, relapsing inflammatory skin disease that poses a significant global public health challenge, affecting 1% to 20% of the worldwide population. More importantly, AD is more common in children, with rates several times higher than in adults [1], and it is particularly prevalent in females. AD follows a bimodal distribution, peaking in early childhood and again in middle age [2]. In the Global Burden of Disease Study from 1990 to 2017, AD ranked 15th among all non-fatal diseases and 1st among skin diseases in terms of Disability-Adjusted Life Years (DALYs) [3]. Treatment often requires frequent medical consultations and adherence, and the pathogenesis is complex, involving genetic factors, impaired skin barrier function, immune dysregulation, and environmental triggers [4]. The heterogeneity of AD is evident in its endogenous and exogenous phenotypes [5], and the differences exist between children and adults [6], and among various ethnic groups [7].

Current treatment recommendations internationally suggest a tiered approach based on disease severity. The primary goals of AD treatment are to minimize triggers, alleviate symptoms, prevent relapses, reduce complications, and improve quality of life. Mild cases may be treated with topical calcineurin inhibitors (TCI) or topical corticosteroids (TCS). Moderate cases often require continued treatment with TCS/TCI after initial control of lesions, while severe cases may necessitate systemic immunosuppressants, biologics, or phototherapy. However, these treatments carry potential limitations and adverse effects, such as skin atrophy, osteoporosis, and infections, underscoring the need for safer alternative therapies.

**Fig. 1:**
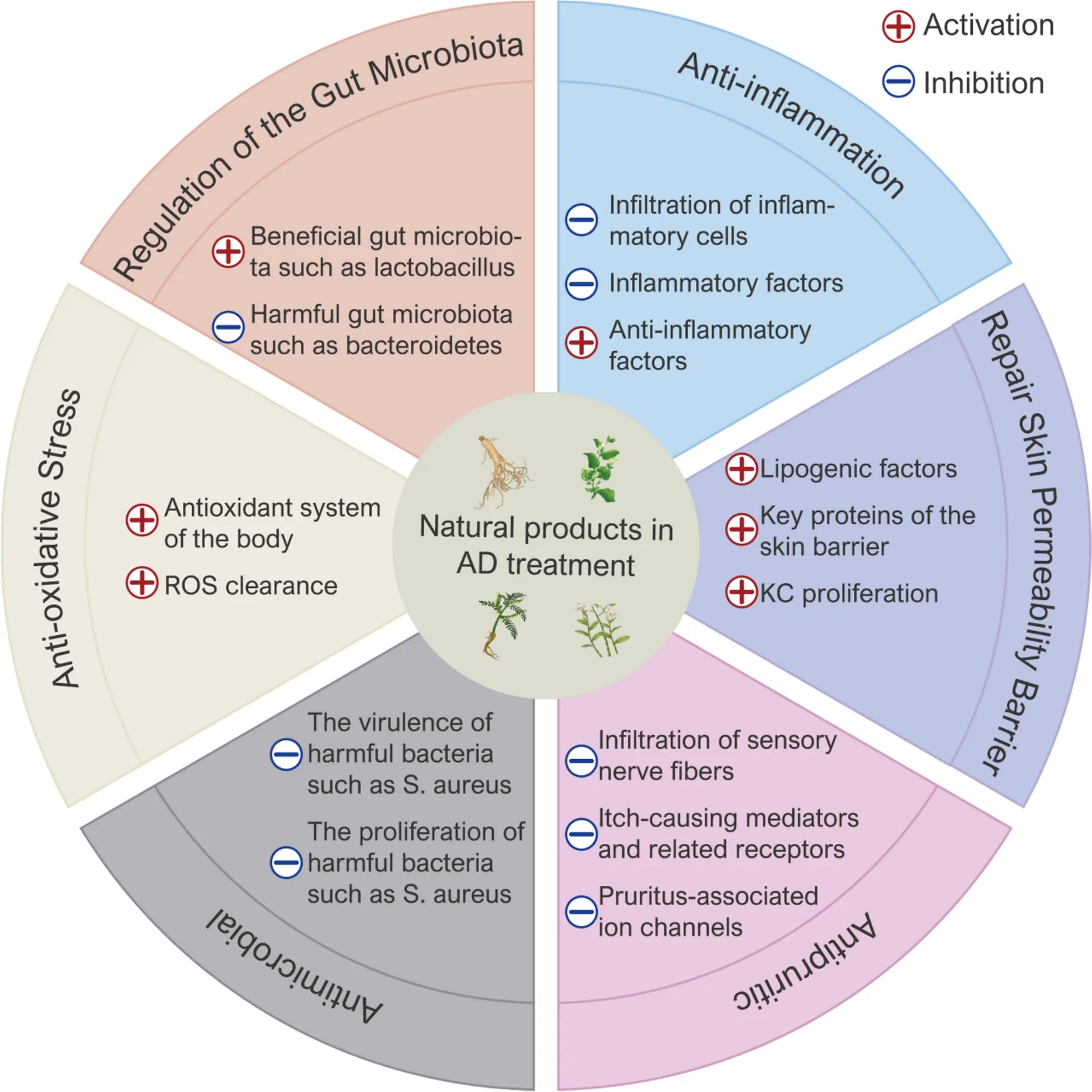
Natural products in AD treatment based on different mechanisms

Natural ingredients have garnered extensive attention due to their natural properties and minimal adverse effects. Their therapeutic efficacy in the field of dermatology has been repeatedly validated [8]. Scientific research has demonstrated that certain traditional Chinese herbal medicine (CHM), such as *Scutellaria baicalensis* Georgi (*S. baicalensis*) and *Artemisia annua* L. (*A. annua*), exhibit anti-inflammatory and immunomodulatory effects by inhibiting the production of inflammatory mediators, modulating inflammation-related pathways, or regulating immune cell functions. Additionally, herbs like *Curcuma longa* L. (*C. longa*) exert antioxidant effects by regulating the antioxidant enzyme system and scavenging free radicals. These mechanisms collectively contribute to the control of AD symptoms, offering higher safety and patient compliance [9]. With the ongoing exploration and application of natural CHMs, a substantial body of experimental research has revealed the advantages and potential of various herbal components in the treatment of AD. This article reviews domestic and international studies on the use of CHM in AD treatment and comprehensively summarizes its efficacy and advantages in relation to the pathogenesis of AD, with the purpose of providing a reference for the development of herbal components as safer adjunctive therapeutic agents or supplements for AD (Fig. 1).

## Limitations of current treatment strategies for AD

Currently, several treatment strategies have been developed for AD. A crucial aspect of AD treatment is the maintenance of skin function. Therefore, the foundation of typical AD treatment is skin care, which includes maintaining skin moisture to reduce dryness and pruritus [10, 11]. Inflammatory factors are the main determinants of the severity and progression of AD. In recent years, clinical treatments for AD have mainly included TCS, TCI [12] and immunotherapies [13]. In addition to chronic skin inflammation, persistent itching is the most prominent and difficult-to-control symptom for AD patients [14]. Antihistamines are the first choice for suppressing itching, but long-term use of these drugs may lead to adverse effects such as palpitations and skin bleeding [15], and studies have shown that itching also has histamine-independent subtypes. Phototherapy is another commonly employed modality for AD [16]. Phosphodiesterase 4 (PDE-4) inhibitors are a class of non-glucocorticoid drugs used for the treatment of inflammatory diseases, including crisaborole [17], roflumilast [18], and difamilast [19]. The most common adverse reactions are mild to moderate burning or stinging sensations at the application site [17–20]. Currently, novel targeted agents, including biologics and Janus kinase inhibitors (JAKis), are increasingly favored over traditional immunosuppressants for the treatment of AD. Owing to the complex pathogenesis and ethnic differences, developing targeted therapies remains highly challenging [20–27]. To date, the FDA has approved several novel targeted drugs for AD, including dupilumab (anti-IL-4Rα) in 2017, tralokinumab (anti-IL-13) in 2021, lebrikizumab (anti-IL-13) in 2024, nemolizumab (anti-IL-31Rα) in 2024, as well as JAKis targeting JAK1 (upadacitinib in 2022 and abrocitinib in 2022) or JAK1/2 (ruxolitinib cream in 2021). Biologics are generally administered subcutaneously; therefore, injection-site reactions are specific biologic-related adverse events, including swelling, erythema, pruritus, and pain around the injection site [28]. In addition, common adverse events (AEs) associated with biologics include conjunctivitis, nasopharyngitis, and upper respiratory tract infections. Frequently reported serious AEs include infections, Ménière’s disease, and acute pancreatitis [29–34]. JAKis interfere with the JAK-signal transducer and activator of transcription (STAT) signaling pathway, which plays a central role in the immunopathogenesis of AD [35]; however, they may suppress the body’s immune defense and impair its ability to combat pathogens. Previous studies have shown an association between infections and JAKis, with related infectious AEs including herpes virus infection, upper respiratory tract infection, and tuberculosis [36, 37]. Furthermore, several JAKi-specific AEs warrant attention: gastrointestinal perforation, precancerous lesions, pulmonary thrombosis, and venous thromboembolism [38–40]. Due to their low molecular weight, JAKis can be administered orally; however, oral JAKis carry a boxed warning [41]. In contrast, current clinical trials indicate that topical JAKis have a favorable safety profile. With the widespread clinical application of these drugs, continuous pharmacovigilance regarding the risk of AEs associated with biologics and JAKis remains essential. Table 1 presents the specific mechanisms and limitations of the current therapeutic strategies for AD.

**Table 1:** Current therapeutic strategies for AD

Therapeutics	Mechanism of action	Therapeutic effects	Limitations or side effects	Refs.
Emollients	Hydration and improvement of skin hydration	TEWL (↑)	Inability to resist S. aureus colonization and ineffectiveness in reducing the severity of AD	[10, 11]
Immunosuppressants	Affect the interaction between antigen-presenting cells and T lymphocytes, and inhibit T cell proliferation and the synthesis of pro-inflammatory cytokines	Proinflammatory cytokines (↓) Pruritus (↓)	Hypertension, hepatorenal toxicity, hirsutism, gingival hyperplasia, bone marrow suppression, chronic immunosuppression	[13]
TCS	Attenuate the transcription of pro-inflammatory cytokines while inducing the transcription of anti-inflammatory mediators	Proinflammatory cytokines (↓)	Cutaneous atrophy, alterations in the HPA axis, rosacea, lymphoma, and the development of other malignancies	[12]
TCI	Inhibition of calcineurin dephosphorylation reduces T cell activation	Proinflammatory cytokines (↓)	Burning sensation, stinging, and pruritus	
Antihistamines	Inhibiting histamine release and breaking the itch-scratch cycle	Pruritus (↓)	Lack of anti-inflammatory effects and inability to treat the underlying disease itself	[15]
Phototherapy	NbUVB (311 nm) blocks the function of antigen-presenting cells and inhibits the production of inflammatory cytokines by KCs	Skin barrier (↑) Proinflammatory cytokines (↓) Pruritus (↓)	Erythema is induced and may be exacerbated by overheating and sweating	[16]
	UVA1 (340–400 nm) inhibits epidermal LCs and eosinophil (EOS)		Eczema, vesicles, hyperpigmentation, and skin aging	
Phosphodiesterase-4 inhibitors	Inhibiting the degradation of cAMP, which regulates inflammation	Proinflammatory cytokines (↓)	Burning and stinging	[20]
Dupilumab	Blocking the signaling pathways of IL-4 and IL-13	Proinflammatory cytokines (↓) Pruritus (↓)	Injection site reactions, conjunctivitis and blepharitis, oral herpes infections, and upper respiratory tract infections, with poor medication adherence	[21, 22]
Nemolizumab	Blocking the signaling pathways of IL-31	Proinflammatory cytokines (↓) Pruritus (↓)	COVID-19, nasopharyngitis, atopic dermatitis, upper respiratory tract infection	[29]
Tralokinumab	Blocking the signaling pathways of IL-13	Proinflammatory cytokines(↓) Pruritus(↓) Skin barrier (↑)	Eczema herpeticum, generalized exfoliative dermatitis, alopecia, skin exfoliation, and blepharitis	[33]
Lebrikizumab	Blocking the signaling pathways of IL-13	Proinflammatory cytokines(↓) Pruritus(↓)	Injection site pain, conjunctivitis, and rash	[34]
JAK inhibitors	Blocking the JAK family to disrupt the JAK-STAT signaling pathway that regulates immune responses	Th2 axis cytokines(↓)	Increased susceptibility to infections, elevated levels of creatine phosphokinase, potential risk of malignancy, and thrombotic events	[36]

## Progress of natural ingredients in AD treatment based on different mechanisms

**Fig. 2:**
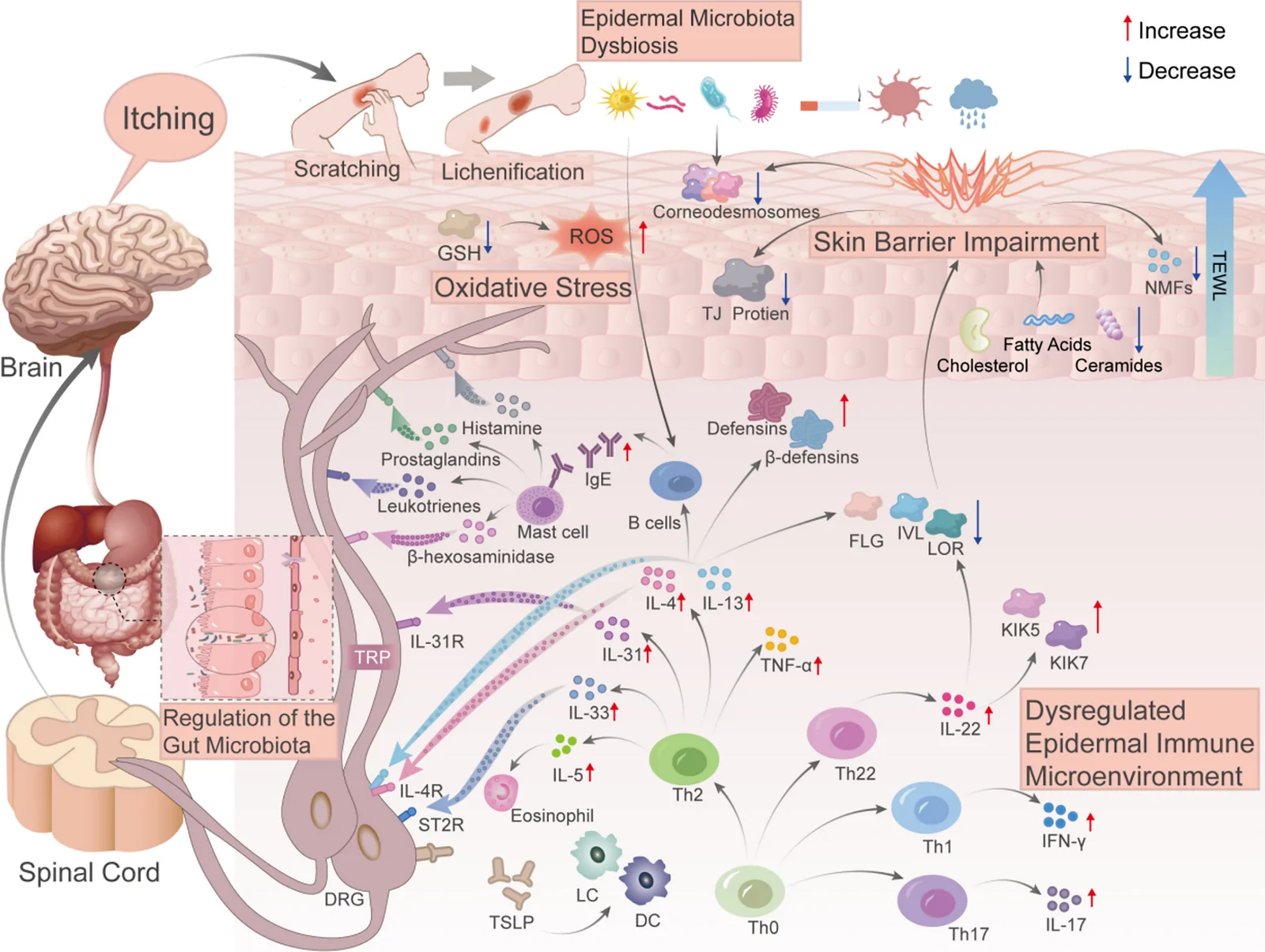
The sketch of the pathogenesis of AD. Dysfunction of the immune microenvironment in the epidermis leads to the release of inflammatory factors, chemokines, and pruritogenic mediators by various effector cells, thereby influencing itch sensation and compromising the epidermal permeability barrier. This cascade triggers scratching behavior and subsequent skin tissue damage. Genetic mutations or external injuries impair the skin barrier function, increasing TEWL and rendering the skin more susceptible to external stimuli. This heightened sensitivity often disrupts the epidermal microbiota balance, further promoting the release of pro-inflammatory factors. Gut dysbiosis affects immune cell activity and cytokine secretion, thereby modulating the initiation and progression of inflammatory responses. OS disrupts the epidermal immune microenvironment by inducing excessive immune cell differentiation and causing lipid peroxidation in KC cell membranes. These processes contribute to epidermal cell damage and death, exacerbating the severity of AD

Regarding the pathogenesis of AD, two hypotheses have been proposed: "inside-out" and "outside-in." The "inside-out" hypothesis suggests that skin inflammation caused by altered immune responses precedes and predisposes to the loss of normal skin barrier function. In this view, AD results from immune system dysregulation, primarily due to the activation of Th, which leads to increased release of pro-inflammatory factors that suppress the expression of barrier-related proteins in keratinocytes (KCs). Pruritic factors induce itching, resulting in scratching that further damages the skin barrier function. Conversely, the "outside-in" hypothesis posits that skin barrier disruption occurs prior to skin inflammation and is likely to trigger it. This means that genetic mutations or external insults impair skin barrier function, leading to dry skin and thinning of the stratum corneum(SC), which makes the skin more sensitive to external stimuli. This sensitivity activates immune cells and triggers the release of a series of pro-inflammatory factors, resulting in inflammation [42]. Additionally, this can lead to an imbalance in the skin microbiome, further exacerbating inflammation [43]. A unified "inside-outside-inside" hypothesis has also been proposed, which suggests that changes in immune reactivity lead to secondary barrier dysfunction, subsequently promoting skin inflammation due to the activation of type 2 immune responses [44]. Regardless of the sequence, skin barrier dysfunction, itching, microbial infection, and the release of pro-inflammatory factors are all critical components in the development of AD (Fig. 2). Targeted therapies can be directed at these components to achieve effective treatment outcomes.

### Anti-inflammatory

#### Immune dysregulation

Inflammation is the primary factor influencing the severity and duration of AD, making anti-inflammatory treatment a crucial step in managing AD. Current knowledge indicates that the robust activation of Th2 and Th22 immune responses in the skin and serum plays a key role in the immunopathogenesis of AD, particularly during the acute phase when cluster of differentiation (CD)4 + T cells polarize towards the Th2 phenotype, leading to an imbalance in the Th1/Th2 ratio. This is followed by the variable activation of Th1 and Th17 in the chronic phase, resulting in increased release of pro-inflammatory factors [45, 46]. These pro-inflammatory cytokines mainly include IL-4, IL-5, IL-13, IL-31, IL-22, tumor necrosis factor-α (TNF-α), interferon-γ (IFN-γ), and IL-17 [47]. IL-4 and IL-13 can reduce the expression of FLG, loricrin (LOR), involucrin (IVL), and lipid components in the skin barrier, leading to epidermal barrier dysfunction [48, 49]. Moreover, downstream signaling of IL-4 and IL-13 regulates the expression of innate immune response genes, including defensins and β-defensins, enhancing susceptibility to skin infections, such as S. aureus [50–52]. IL-4 can also activate B cells to produce Immunoglobulin E (IgE) antibodies, and when these antibodies interact with the specific receptor Fcepsilon RI on mast cells, they activate a signaling cascade, ultimately leading to increased intracellular Ca2 + , mast cell degranulation, and the release of allergic mediators (histamine, prostaglandins, β-hexosaminidase, and leukotrienes) [53]. IL-5 induces EOS recruitment [54], while IL-31 is associated with the occurrence of itching [55], leading to scratching and further damage to the skin barrier. Additionally, IL-22 reduces the expression of trichocyte keratin filaments by activating the Janus kinase (JAK)1-signal transducers and activators of transcription (STAT)3 pathway in KCs and can increase the expression of skin barrier-degrading enzymes kallikrein (KLK) 5 and KLK7, thereby causing destructive skin barrier dysfunction through lipid disruption [56]. This barrier dysfunction allows allergens or microbes and other foreign irritants to penetrate more easily, exacerbating inflammation. Besides the aforementioned pro-inflammatory cytokines, thymus and activation-regulated chemokine (TARC/CCL17), macrophage-derived chemokine (MDC/CCL22), and regulated upon activation normal T cell expressed and secreted (RANTES/CCL5) are also inflammatory chemokines involved in Th2-dominated immune responses [57]. They are expressed in KCs stimulated by inflammatory cytokines to attract Th2 cell infiltration into skin lesions [58]. LCs and DCs participate in the Th2 inflammatory process by presenting allergens, and LCs triggered by thymic stromal lymphopoietin (TSLP) can promote the polarization of Th0 towards Th2 and increase the production of TNF-α and IL-4 [59, 60]. Th17 cells can promote the development of autoimmunity, inflammation, and allergy [61], and Th17 cell activation is also a pathogenic factor in AD [62]. Certain natural medicinal materials and Chinese herbal formulas have been shown to balance Th1/Th2, Th17/Th22 cell differentiation, inhibit the release of pro-inflammatory factors, and thus improve clinical skin lesions in AD.

#### The anti-inflammatory effects of natural ingredients in AD treatment

In the natural product therapy for atopic dermatitis, single bioactive compounds represent the most direct and readily elucidatable class of material basis for investigating their anti-inflammatory mechanisms. Single compounds possess well-defined chemical structures and high purity, which enables the precise elucidation of their interactions with key targets in inflammatory signaling pathways, such as NF-κB, MAPK, and JAK-STAT. Studies have demonstrated that magnolol **(1)** [63], evodiamine **(2)** [64], α-cubebenoate **(3)** [65], glycyrrhizic acid (GA) **(4)** [66], luteolin **(5)** [67], and luteolin 7-methyl ether **(6)** [68] can differentially modulate Th1/Th2/Th17 immune responses via distinct pathways, thereby reestablishing immune homeostasis and conferring therapeutic efficacy. Furthermore, the specific mechanisms of action of several other compounds have been preliminarily investigated, although these studies are relatively limited compared to those on the aforementioned compounds. For example, daphnetin **(7)**, a coumarin derivative derived from *Daphne koreana* Nakai, has been shown to inhibit the development of 2,4-dinitrochlorobenzene (DNCB)-induced AD-like lesions in BALB/c mice by reducing levels of IgE and inflammatory cytokines. This effect has been further validated in vitro, revealing inhibition of mast cell degranulation via suppression of the MAPK/ERK/p38 signaling pathway and regulation of the Th1/Th2 balance [69]. Baicalin **(8)**, a flavonoid from *S. baicalensis*, has been shown to alleviate skin inflammation by modulating the NF-κB and JAK/STAT signaling pathways, although this represents only one aspect of its anti-AD mechanisms [70]. Beyond these multi-target components, other herbal compounds have also demonstrated promising anti-AD effects, even though their broader mechanisms remain underexplored. For instance, mangiferin **(9)** has been reported to suppress the expression of chemokines and Th2 cytokines in AD-like mice and TNF-α/IFN-γ co-stimulated HaCaT cells [71]. Similarly, Prim-O-glucosylcimifugin (POG) **(10)**, the most abundant chromane in *Saposhnikovia divaricata* (Turcz.) Schischk., has been found to significantly inhibit Th2 cell differentiation in vitro, with no significant effects on the differentiation of Th1, Th17, and Treg cells. This has been verified in the Th2 immune response of AD mice induced by MC903 [72]. Furthermore, notably, notoginsenoside R1 **(11)** has been demonstrated to suppress inflammation and alleviate AD symptoms by inhibiting the NF-κB signaling pathway and NLRP3 inflammasome activation [73].

However, in addition to single components, specific bioactive fractions in traditional Chinese medicine (TCM) have also shown significant therapeutic efficacy. These fractions, such as total flavonoids and saponins, represent defined categories of phytochemicals extracted from TCM. While they do not possess a single defined chemical structure, they consist of well-characterized compositional profiles. For instance, the essential oils of *A. annua* [74], *Rosmarinus officinalis* L. [75], *Lavandula angustifolia Mill* [76], *Mentha arvensis* [77] have been demonstrated to suppress immune cell differentiation and downregulate the expression of key pro-inflammatory cytokines and chemokines. These anti-AD activities have been confirmed through in vivo studies using AD mouse models or in vitro assays in RAW 264.7 macrophages and HaCaT keratinocytes. Beyond these essential oil components, the total flavonoid compounds from *Hippophae rhamnoides* L. (TFH) also have shown therapeutic potential through comprehensive in vivo and in vitro studies [78]. In addition, the sterol ester fraction obtained via CO_2_ supercritical extraction from *Stellariae Radix* has demonstrated efficacy in AD treatment by reducing pro-inflammatory cytokines in an AD mouse model [79].

Certain medicinal substances, although derived from a single plant or plant part, contain a complex mixture of bioactive compounds that exert broad pharmacological effects through synergistic interactions. For instance, the water extract of Terminalia chebula (TC) [80], *Isatis tinctoria* L. [81], and the ethanol extract of *Grifola frondosa* [82] have demonstrated in vivo effects on improving AD lesions and in vitro effects on suppressing inflammatory factors. Similarly, the major constituents of *Pyrus ussuriensis Maxim.* Leaves (PUL)—arbutin, chlorogenic acid, and rutin—may act individually or synergistically to modulate AD pathogenesis [83]. Other TCM components, such as *Veronica persica* ethanol extract [84], *Lycium barbarum* leaves ethanol extract [85], red ginseng (RGE) water extract [86], have both shown good anti-AD effects in these two AD models, with the latter and PUL also reducing the levels of splenocyte inflammatory cytokines. *Curcuma longa* leaves and pseudostems (CLHW) extract has exhibited similar effects. However, it more robustly reduced AD-related cytokine mRNA expression in in vivo spleen and lymph node tissues [87]. Additionally, the water extract of *A. annua* has been proven to inhibit the severity of DNCB-induced AD in mice by suppressing Th2-type inflammatory responses [88].

In summary, these studies indicate that the administration of natural ingredients, either systemically or topically, can regulate the immune system by inhibiting the infiltration of inflammatory cells, blocking the production or action of cytokines, or stimulating the production of anti-inflammatory cytokines such as IL-10. This regulation helps to balance the Th1/Th2/Th17/Th22 immune responses, thereby preventing the development of AD. Numerous studies have demonstrated that natural ingredients exert their anti-atopic effects primarily by regulating signaling pathways such as NF-κB and JAK/STAT, thereby inhibiting skin inflammation. Table 2 summarizes the natural components that exert anti-inflammatory effects in the treatment of AD.

**Table 2:** Summary of the natural products that exert anti-inflammatory effects in the treatment of AD

Natural products	Source	Study	Dosing regimen	Positive control drug	AD model	Dermatitis score	Ear thickness	Epidermal thickness	Inflam-matory cell infiltration	Inflammation cytokines and chemokines (protein)	Inflammation cytokines and chemokines (mRNA)	IgE	Influence of Relevant Mechanistic Pathways	Refs.
Magnolol	*Magnolia officinalis*	In vivo	Intraperitoneally, 5/10 mg/kg, every other day from day 7 to day 42	DXM, intraperitoneally, 10 mg/kg	DNCB- treated BALB/c Mice	—	↓	—	↓	—	IL-13, IL-17A, IFN-γ, IL-12A, TARC, IL-8, IL-6↓	↓	Inhibition of Th2/Th17 differentiation	[63]
Evodiamine	*Evodia rutaecarpa Bentham* (Rutaceae) fruits	In vivo	Intraperitoneally, 10 and 20 mg/kg, every other day from day 19 to day 48	DXM, intraperitoneally, 10 mg/kg	DNCB- treated BALB/c Mice	—	↓	↓	↓	—	IL-4, IL-13, IL-17A, INF-γ, IL-12A, IL-22, IL-6, IL-8↓	↓	Inhibition of Th2/Th17/Th1 cytokines	[64]
α-cubebenoate	*Schisandra chinensis*	In vivo	Intraperitoneally, 0.5/1 mg/kg, every other day from day 19 to day 48	DXM, intraperitoneally, 10 mg/kg	DNCB- treated BALB/c Mice	↓	↓	↓	↓	—	IL-4, IL-13, INF-γ, IL-17A, CCL17, CCL22↓	↓	Regulation of Th2/Th17/Th1 immune balance	[65]
Glycyrrhizic acid	Glycyrrhiza	In vivo	Intraperitoneally, 25/50 mg/kg, every other day from day 3 to day 17	DXM, intraperitoneally, 2 mg/kg	MC903- induced C57BL/6 mice	↓	↓	↓	↓	IL-4, IFN-γ, TNF-α, TSLP↓; FLG, LOR↑	—	↓	Regulation of Th1/Th2/Th1 immune balance-	[66]
Luteolin	Medicinal plants worldwide	In vivo	Topically, 0.5%, 100uL, daily from day 1 to day 13	DXM, topically, 0.05%, 100uL	DNFB- treated Kunming mice	↓	—	↓	↓	IL-4, IL-6, TNF-α, IL-17↓; FLG, IFN-γ↑	—	—	Inhibition of Th2/Th17 cytokines and enhancement of Th1 cytokines	[67]
Luteolin 7-Methyl	*Wikstroemia ganpi*	In vitro	10 μM	—	PI-induced RBL-2H3 Cells	—	—	—	—	—	IL-4↓	—	—	[68]
		In vitro	12.5/50 μM	—	TNF-α-treated HaCaT Cells	—	—	—	—	—	IL-6, GM-CSF, G-CSF, TRPV1, IL-31↓	—	—	
Daphnetin	*Daphne koreana* Nakai	In vivo	Intragastrically, 50/100 mg/kg, daily from day 1 to day 21	DXM, orally, 2 mg/kg	DNCB- treated BALB/c Mice	↓	—	↓	↓	IL-4, IL-5, IL-9, IL-13, IL-33, TSLP↓	—	↓	Inhibition of Th2 cytokines	[69]
		In vitro	16.8/56/168 µM	Keto fumarate, 30 μM	DNP-BSA-induced RBL-2H3 Cells					Histamine, IL-4, IL-6, IL-13, MIP-1α, INF-α↓	—	—	Inhibition of MAPKs signaling pathway, and regulation of Th1/Th2 immune balance	
Baicalin	*Scutellaria baicalensis* Georgi	In vivo	Intragastrically, 50/100/200 mg/kg, daily from day 15 to day 28	Abrocitinib, intragastrically, 7.5 mg/kg	DNCB- treated BALB/c Mice	↓	↓	↓	↓	IL-4, TSLP, Histamine↓; FLG, LOR, IFN-γ↑	—	↓	Regulation of the Th1/Th2 balance, modulation of gut dysbiosis, inhibition of of NF-κB and JAK/STAT pathways	[70]
Mangiferin	mango tree	In vivo	Intragastrically, 10/50/100 mg/kg, daily from day 8 to day 28	Hydrocortisone, topically, 1%	DNCB- treated BALB/c Mice	↓	—	↓	↓	—	TSLP, TARC, IL-13, IL-5, IL-4↓	—	Inhibition of the AKT/NF-κB/STAT1 and MAPKs signaling pathways	[71]
		In vitro	25/50 μM	—	TNF-α/IFN-γ- stimulated HaCaT Cells	—	—	—	—	—	IL-4, IL-5, IL-13, TARC, TSLP, RANTES, TNF-α, IL-1β, IL-6↓	—	Inhibition of the AKT/NF-κB signaling pathways	
Prim-O-glucosylcimifugin	*Saposhnikovia divaricata* (Turcz.) Schischk	In vivo	Topically, 2.5/5.0/7.5 mg/mL, 20 uL, daily from day 1 to day12	Halcinonide, topically, 0.1%, 20 uL	MC903-induced C57BL/6 mice	↓	↓	↓	↓	—	TSLP, IL-13, IL-5, IL-4↓	—	Enhancement of the expression of p-LCK	[72]
		In vitro	2/5/10 mol/L	—	TSLP- induced HaCaT cells						IL-13, IL-4↓	—	Inhibit Th2 cell differentiation	
Notoginsenoside R1	*Panax notoginseng*	In vitro	0.1/1/10 μM	—	LPS- stimulated RAW264.7 macrophages cells	—	—	—	—	TNF-α, IL-1β, IL-6, iNOS, COX2, NLRP3, ASC, Caspase1↓	iNOS, COX2↓	—	Inhibition of NF-κB/NLRP3 inflammation signaling pathway	[73]
A.annua essential oil	*Artemisia annua* L	In vivo	Topically, 3/5/10%, daily from day 15 to day 28	Hydrocortisone butyrate cream, topically	DNCB- treated BALB/c Mice	↓	—	↓	↓	—	TNF-α, IL-1β, IL-6 ↓; IL-10↑	↓	Inhibition of MAPK/NF-κB signaling pathway	[74]
Rosemary essential oil	*Rosmarinus officinalis* L	In vivo	Topically, 1/2/4%, Twice daily from day 1 to day 17	DXM, topically	DNCB- treated Kunming mice	—	—	↓	↓	IL-6, TNF-α, IL-4↓	—	—	Inhibition of JAK/STAT/NF-κB signaling pathway	[75]
Lavender essential oil	*Lavandula angustifolia* Mill	In vivo	Topically, 2/4/8%, Twice daily from day 1 to day 17	Dexamethasone acetate cream, topically	DNCB- treated Kunming mice	—	—	↓	↓	TNF-α, IL-6, FOXP3, IL-6, IL-17A, RORγt↓	—	—	Inhibition of STAT3/RORγt pathway in Th17 cell differentiation	[76]
*Mentha arvensis* Essential Oil	*Mentha arvensis*	In vivo	Topically, 0.1/3%, daily from day 7 to day 21	DXM, orally, 1 mg/kg	DNCB- treated BALB/c Mice	↓	↓	↓	↓	—	—	—	—	[77]
		In vitro	12.5/25/50/100 μg/mL	—	LPS-induced RAW 264.7 Macrophages cells	—	—	—	—	NO, PGE2, iNOS, COX-2, IL-6, IL-1β↓	iNOS, COX-2, IL-6, IL-1β↓	—	Inhibition of ERK/NF-κB signaling pathway	
		In vitro		—	LPS-induced HaCaT cells	—	—	—	—	NO, PGE2, iNOS, COX-2, IL-6, IL-1β↓	—	—	—	
Total flavonoids of *Hippophae rhamnoides*	Sea buckthorn	In vivo	Topically, 5/10 mg/kg, daily from day 7 to day 14	DXM cream, topically, 1 mg/kg	MC903- treated C57BL/6 mice	↓	↓	↓	↓	FLG↑	IL-4, IFN-γ, TNF-α, TSLP↓	—	—	[78]
		In vitro	1.25/2.5/5.0 mg/L	—	IFN-γ/TNF-α- induced HaCaT cells	—	—	—	—	IL-1α, IL-1β, MCP-1, MCP-3, MDC, PDGF-BB, TARC, IL-6↓	—	—	Inhibition of MAPK/NF-κB signaling pathway	
Stellariae Radix lipid component	Stellariae Radix	In vivo	Topically, 15、30、60、120、240 mg/g, daily from day 15 to day 18	Compound dexamethasone acetate cream, topically, 0.75 mg/g	DNCB- treated ICR Mice	↓	↓	↓	↓	IFN-γ, IL-1β, TNF-α, NLRP3↓	IL-1β, TNF-α, CXC10, IL-12↓	↓	Activating the AMPK signaling pathway in BMDMs to inhibit NLRP3-mediated release of pro-inflammatory cytokines	[79]
*Terminalia chebula Retz.* Extract		In vivo	Orally, 30/100/300 mg/kg, daily from day 9 to day 22	DXM, orally, 3 mg/kg	DFE-induced NC/Nga mice	↓	↓	—	↓	Histamine, MDC, TARC, RANTES, TSLP↓	—	↓	—	[80]
		In vitro	50/100/300 μg/ml	—	IFN-γ/TNF-α- stimulated HaCaT cells	—	—	—	—	MDC, RANTE, IFN-γ, MCP-1, IL-6↓	IFN-γ, MCP-1, IL-6, IL-8↓	—	Inhibition of STAT1/3 and NF-κB signaling pathway	
*Isatis tinctoria* L. water extract		In vivo	Orally, 100/200 mg/kg, daily from day 14 to day 29	DXM, orally, 1 mg/kg	DNCB- induced BALB/c Mice	↓	—	↓	↓	TNF-α↓	TNF-α, IL-6, IL-13↓	↓	Inhibition of MAPK/NF-κB signaling pathway	[81]
		In vitro	100/200/400 μg/mL	—	TNF-α/IFN-γ- induced HaCaT cells	—	—	—	—	R—TES, TARC, MDC, MCP-1, MIP-3α↓	R—TES, TARC, MDC↓	—	Inhibition of NF-κB nuclear translocation	
*Grifola frondosa* ethanolic extract		In vivo	Topically, 20 μL, 1 mg/mL, Every other day from day 7 to day 28	Ceramide, topically	DNCB- induced BALB/c mice	—	↓	↓	↓	IgG2a↓	IFN-γ, IL-4, IL-13, IL-31, IL-17, IL-22↓	↓	Inhibition of Th2/Th17/Th1/Th22 cytokines	[82]
		In vitro	0.25/0.5/1 mg/mL	Cyclosporine A, 1 mg/mL	TNF-α/IFN-γ- induced HaCaT cells	—	—	—	—	—	TNF-α, IL-6, IL-1β, CCL17↓	—	Inhibition of MAPK signaling pathway	
*Pyrus ussuriensis Maxim.* leaves extract		In vivo	Topically, 1/2.5%, 3 times a week from week 5 to week 11	DXM, topically, 0.1%	DNCB- induced NC/Nga mice	↓	—	—	—	—	—	↓	—	[83]
		In vitro	1/10/100 μg/mL	—	TNF-α- induced HaCaT cells	—	—	—	—	IL-6, IL-1β↓	—	—	—	
		In vitro	0.5/2/8 μg/mL	—	Splenocytes isolated from AD mice	—	—	—	—	IL-4, IL-13↓	—	—	—	
*Veronica persica* Ethanol Extract		In vivo	Orally, 200 mg/kg, daily from day 4 to day 17	DXM, orally, 3 mg/kg	DNCB- induced NC/Nga mice	↓	—	—	↓	Histamine↓	IL-6, IL-13, IL-31R, CCR3, TNFα↓	↓	—	[84]
		In vitro	—	—	Splenocytes isolated from AD mice	—	—	—	—	IL-4, IL-5, IL-13↓ IFN-γ↑	—	—	Regulation of Th1/Th2 immune balance。	
		In vitro	400 μg/mL	DXM, 20 μM	TNF-α/IFN-γ- induced HaCaT cells	—	—	—	—	NRF2, HO-1 ↑	IL-6, IL-13, CXCL10↓	—	Upregulation of Nrf2/HO-1 expression	
*Lycium barbarum* leaf extracts		In vivo	Orally, 1%, feed ad libitum from day 1 to day 49	—	DNCB- induced NC/Nga mice	—	—	↓	↓	TNF-α, IL-6, IL-4↓	—	↓	—	[85]
		In vitro	15.6/125/1000 μg/mL	—	TNF-α- induced HaCaT cells	—	—	—	—	—	TARC, MDC↓	—	—	
Korean red ginseng water extract		In vivo	Orally, 100/200/400 mg/kg, daily from week 1 to week 6	—	DNCB- induced BALB/c Mice	↓	—	↓	↓	Ceramidase, TARC↓; FLG↑	TSLP, IL-6, TNF-α↓	↓	Inhibition of the MAPK signaling pathway and expression of Th2 cytokines and chemokines	[86]
		In vitro	250/500/1000 μg/ml	DXM, 100 μM	TNF-α/IFN-γ- induced HaCaT cells	—	—	—	—	MDC, TARC↓	—	—	—	
*Curcuma longa* L. leaves and pseudostems extract		In vivo	Orally, 50/100/200/400 mg/kg, daily from week 3 to week 5	Cetirizine hydrochloride tablet, orally, 5 mg/kg	DNCB- induced BALB/c Mice	↓	↓	↓	↓	IL-4、IL-5、IL-13↓	TSLP, IL-25, IL-33;IL-4, IL-10, IL-13, TARC, MDC, TNF-α, IFN-γ↓	↓	—	[87]
*Artemisia annua* water extract		In vivo	Orally, 1.5/3/6 g/kg, daily from day 7 to day 19	DXM, orally, 1.5 mg/kg	DNCB- induced BALB/c Mice	↓	↓	—	↓	IL-4, IL-13, NF-κB↓	IL-4, IL-6, IL-13, IL-17, TNF-α, TSLP↓	↓	Inhibition of MAPK/NF-κB signaling pathway and Th2 type inflammatory response	[88]

—, Not Applicable or Not Determined

### Repair of skin permeability barrier

#### Skin barrier dysfunction

From an etiological perspective, the pruritus and erythema associated with AD are attributed to defects in the barrier function. The functionality of the permeability barrier depends on its structural components, which require an appropriate epidermal structure to maintain normal permeability barrier homeostasis. The outermost layer of the epidermis, the SC, is composed of flat KCs, which are the end products of KCs differentiation [89]. These KCs are anucleate, and all their organelles and cytoplasmic membranes are replaced by a single layer of ω-esterified long-chain fatty acids and ceramides. Ceramides are covalently bound to a protein mixture on the outer surface of the cornified envelope (CE), including IVL, LOR, and others [90, 91]. Cellular contacts between adjacent KCs in the SC are established through corneodesmosomes [44, 92]. Impaired barrier function in dry, atopic skin is characterized by increased transepidermal water loss (TEWL) and decreased water-binding capacity, primarily due to changes in the levels of lipid components in the SC. Studies have shown that barrier abnormalities are associated with selective reductions in one or two of the three main intercellular lipids, including cholesterol, free fatty acids, and ceramides [93]. Three distinct peroxisome proliferator-activated receptors (PPARs) have been identified in mammals: PPAR-α, PPAR-β/δ, and PPAR-γ. PPAR-α is an important regulator of lipid metabolism [94, 95], and during epidermal differentiation, the expression of PPAR-α can increase the synthesis of cholesterol and ceramides in KCs [96, 97].

Among the many epidermal proteins, FLG has been the most extensively studied. FLGs are key proteins in the terminal differentiation of epidermal KCs and, together with SC intercellular lipids, form the skin barrier. Additionally, the degradation of FLG proteins produces pyrrolidone carboxylic acid, free amino acid, and urocanic acid, which are essential for the formation of natural moisturizing factors (NMFs) [98] and play a significant role in maintaining the hydration of the SC [99]. Once these FLGs are reduced or absent due to genetic mutations, the FLG-associated SC barrier is compromised [100–102]. This disruption of the SC barrier leads to a decrease in desmosomes, NMFs, tight junction (TJ) proteins, and an increase in pH, thereby increasing the incidence of transdermal allergen exposure and lowering the threshold for skin inflammation [103]. These findings all demonstrate the importance of key components of the skin barrier for normal function. Based on the aforementioned pathogenic mechanisms, the treatment of AD should include strategies that prolong the SC pH at a lower value (over acidification), and the application of serine protease inhibitors, topical Protease-Activated Receptor (PAR) 2 antagonists, PPAR agonists, Antimicrobial Peptides (AMPs) enhancers, or TJ component substitutes. However, these drugs can cause various toxic side effects.

#### The skin repairing effects of natural ingredients in AD treatment

Nowadays, numerous studies have confirmed the potential of natural ingredients to treat AD by repairing the skin barrier. It is known that the activation of PPAR-α via ligand binding can promote the differentiation of KCs and reconstruct the damaged skin barrier. The flavonoids quercetin **(12)** and isorhamnetin **(13)** in Eruca sativa (ES) extract serve as the main PPAR-α active components. Cellular experiments have shown that their activation of PPAR-α ligand binding can promote the restoration of anti-inflammatory and skin barrier-related proteins, thereby improving skin barrier function [104]. Local application of valencene **(14)** from Cyperus rotundus also achieved similar effects [105]. It has been reported that, in addition to the aforementioned anti-inflammatory effects, GA **(4)** and baicalin **(8)** can restore FLG and LOR expression in AD-like mice induced by various sensitizers, repair the skin barrier, and further improve AD symptoms in a dose-dependent manner [66, 70]. Regarding the flavonoid component of *Hordeum vulgare* L. (Barley), saponarin **(15)**, studies have only been conducted in vitro, where it significantly induced the expression of hyaluronic acid synthase-3 (HAS-3) and aquaporin-3 (AQP-3) in HaCaT cells, which play an important role in skin barrier repair [106]. The active component of Lobelia chinensis extract (LCE), diosmetin **(16)**, primarily enhances endogenous protease inhibitors and inhibits protease expression. It regulates the SPINK5/LEKTI pathway to modulate the protease cascade involved in skin inflammation, which plays a significant role in skin barrier disruption [107]. These natural components have been proven to be effective in treating AD and repairing the skin barrier, providing strong evidence for further research and the development of novel and effective AD barrier repair therapies.

In addition to the aforementioned single active ingredients, numerous studies demonstrate that orally administered herbal extracts can ameliorate skin barrier dysfunction in murine AD models. Park et al. observed that RGE administration in DNCB-induced AD mice improved dermatitis severity scores, normalized skin pH and hydration levels, and upregulated expression of skin barrier-related proteins [86]. Similarly, PUL treatment enhanced skin hydration while reducing erythema, indicating its potential to preserve epidermal barrier function and modulate AD progression [83]. Additionally, ES treatment significantly enhanced PPAR response element transactivation and stimulated expression of cutaneous barrier-protective proteins [104]. Emerging evidence implicates transient receptor potential vanilloid 3 (TRPV3) in epidermal homeostasis through modulation epidermal growth factor receptor (EGFR) signaling in keratinocytes, particularly during cornification—a critical process in barrier formation [108]. TRPV3 activation also accelerates wound healing by promoting epithelial proliferation [109]. Notably, *Tribulus terrestris* L. extract was identified as the first botanical agent capable of modulating skin barrier-associated ion channels, demonstrating significant TRPV3 activation potential [110]. Subsequent studies revealed that a 70% methanol extract of Spirodelae Herba (SH) exhibits potent TRPV3 agonist activity, supporting its therapeutic application for inflammatory dermatoses [111] and validating ion channel modulation as a novel therapeutic approach for barrier dysfunction in AD. Mechanistic studies show that the chloroform extract of *Fritillaria thunbergii* Bulbus (FT-Cl) upregulates FLG, INV, and AQP-3 expression in TNF-α/IFN-γ-stimulated HaCaT cells. While the ethanol extract (FT-Et) also preserves barrier protein integrity at high concentrations, FT-Cl demonstrates superior efficacy both in vitro and in vivo, with more pronounced anti-inflammatory effects in AD models [112]. Similarly, LCE dose-dependently enhances SPINK5 promoter activity and prevents DNCB-induced barrier impairment by modulating SPINK5 downstream effectors, with active constituents already identified [107]. Notably, Dictamni Cortex (DC) appears in 12.68% of herbal AD formulations, representing the most frequently employed monotherapy [113]. Pharmacological studies confirm that high-dose DC extract (DCE) significantly enhances FLG expression in DNCB-challenged murine skin, with efficacy comparable to Dexamethasone (DXM) [114]. Similarly, the major bioactive components of TFH, when formulated into an emulsion for treatment, not only exhibited anti-inflammatory effects but also significantly upregulated FLG expression. Compared with DXM treatment, which caused marked changes in KC morphology and indistinct cell boundaries, local application of TFH significantly improved these conditions, with regular cell morphology and clear boundaries. This indicates that TFH has a distinct advantage over DXM in skin-barrier repair [78].

To sum up, these studies indicate that interventions targeting epithelial barrier function are preventative and therapeutic approaches for recurrent allergic inflammation in AD. Both systemic and topical applications of natural components can control the development of AD by improving the epidermal permeability barrier function and SC hydration associated with AD. Specifically, current research focuses on repairing the skin barrier through the following aspects. Firstly, enhancing the activity of lipid metabolism regulatory factors such as PPAR during epidermal differentiation to increase the synthesis of lipid components like cholesterol and ceramides in KCs, thereby improving skin epidermal homeostasis. Secondly, upregulating the expression of key proteins such as FLG, LOR, and IVL in epidermal KCs that co-form the skin barrier with lipids. This leads to an increase in NMFs and AQP-3, which control TEWL, regulate pH, and are used for immune modulation. These actions improve skin hydration, restore skin barrier function, and raise the threshold for inflammation. Thirdly, inhibiting the activity of proteases (such as KLK) or/and enhancing the activity of counter-regulatory protease inhibitors (such as endogenous inhibitory factors) to block the degradation of corneodesmosomes, increasing the integrity and compactness of the SC. In addition to the aforementioned approaches, enhancing the function of ion channels related to the skin barrier to promote epithelial KCs proliferation and facilitate skin healing. These studies further suggest that the potential efficacy of natural ingredients in preventing the development of AD can be assessed using non-invasive methods that evaluate the biophysical properties of the SC. Table 3 shows the effective natural products in repairing the skin barrier in AD-like lesions.

**Table 3:** The effective natural products in repairing the skin barrier in AD-like lesions

Natural products	Source	Study	Dosing regimen	Positive control drug	AD model	Dermatitis score	TEWL	Skin hydration	Skin barrier associated proteins	Refs.
Glycyrrhizic acid	Glycyrrhiza	In vivo	Intraperitoneally, 25/50 mg/kg, every other day from day 3 to day 17	DXM, intraperitoneally, 2 mg/kg	MC903-induced C57BL/6 mice	↓	—	—	FLG, LOR↑	[66]
Baicalin	*Scutellaria baicalensis* Georg	In vivo	Intragastrically, 50/100/200 mg/kg, daily from day 15 to day 28	Abrocitinib, intragastrically, 7.5 mg/kg	DNCB-induced BALB/c mice	↓	↓	—	FLG, LOR↑	[70]
TFH	Sea buckthorn	In vivo	Topically, 5/10 mg/kg, daily from day 7 to day 14	DXM cream, topically, 1 mg/kg	MC903-induced C57BL/6 mice	↓	—	—	FLG↑	[78]
Valencene	*Cyperus rotundus*	In vivo	Topically, 50/100 μM, 200 μL, 3 times a week for 10 weeks	DXM, topically, 0.1%, 200 μL	DNFB-induced NC/Nga mice	↓	—	—	FLG↑	[105]
		In vitro	10/50/100 μM	DXM	TNF-α/IFN-γ-induced HaCaT cells	—	—	—	FLG, LOR, IVL↑	
Saponarin	Barley sprouts	In vitro	100 μM	—	TNF-α/IFN-γ-induced HaCaT cells				HAS-3, AQP-3 ↑	[106]
Diosmetin	*Lobelia chinensis*	In vivo	Topically, 0.5%, 200 μL, 3 times a week from week 2 to week 3	—	DNCB-induced SKH-1 hairless mice	↓	↓	↑	FLG, LEKTI ↑ KLK5, DCS1↓	[107]
		In vitro	3/10/30 μM	—	UVB-irradiated HaCaT Cells	—	—	—	SPINK5, LEKTI↑; KLK5, KLK7, PAR2↓	
KRG water extract		In vivo	Orally, 100/200/400 mg/kg, daily from week 1 to week 6	—	DNCB-induced BALB/c mice	↓	↓	↑	FLG↑	[86]
*Lobelia chinensis* extract		In vivo	Topically, 1%, 200 μL, 3 times a week from week 2 to week 3	—	DNCB-induced SKH-1 hairless mice	↓	↓	↑	SPINK5, LEKTI, DSC1, FLG↑; KLK5↓	[107]
*Tribulus terrestris* L. extract		In vitro	100 μg/mL	2-aminoethoxydiphenyl borate, 100 μg/mL	Orai1 or TRPV3-transfected HEK293T cells	—	—	—	Orai1↓; TRPV3↑	[110]
*Spirodela polyrhiza* extract		In vitro	100 μg/mL	2-aminoethoxydiphenyl borate, 100 μg/mL	Orai1 or TRPV3-transfected HEK293T cells	—	—	—	Orai1↓; TRPV3↑	[111]
*Fritillariae Thunbergii* Bulbus-chloroform fraction extract		In vivo	Topically, 10/100 mg/kg, 3 times a week for 5 weeks	—	DNCB-induced BALB/c mice	↓	—	—	FLG, LOR, INV↑	[112]
		In vitro	12.5/25/50 μg/mL	—	TNF-α/IFN-γ-stimulated HaCaT cells	—	—	—	FLG, INV, LOR, AQP-3↑	
Dictamni Cortex extract		In vivo	Orally, 0.6/1.2/2.4 g/kg, daily from day 14 to day 29	DXM, orally, 2.5 mg/kg	DNCB-induced BALB/c mice	↓	—	—	FLG↑	[114]

—, Not Applicable or Not Determined

### Antipruritic

#### Pruritus

AD is one of the most pruritic skin diseases, with itching sensations mediated by the complex interplay between the skin, immune system, and nervous system [115]. The skin contains a sophisticated sensory nerve network, and itching occurs when proteases, cytokines, neuropeptides, and allergens—either exogenous or endogenous triggers—stimulate itch-specific C-fibers at the junction of the epidermis and dermis. Subsequently, these neuronal signals are transmitted to the spinal cord and propagated to the brain, where itching is perceived and scratching is induced [116, 117].

We now understand that itch transmission can be divided into two categories: histamine-dependent and histamine-independent pathways [118]. These two systems, although closely related, seem to exist entirely independently. Their separation begins in the periphery, with each system having its own receptors and skin nerve fibers, and continues into the central nervous system, where each has dedicated nerve tracts and neural structures [118–120]. Transient receptor potential (TRP) channels are ion channels present in KCs and sensory nerves, and several TRP channels have been shown to play a role in mediating atopic itch, with TRPV1, transient receptor potential ankyrin (TRPA) 1, and TRPV3 being the most extensively studied [121, 122]. These channels are activated by various pruritogens, including histamine, serotonin, lipids, and Th2 cytokines [123]. Histamine is a mediator in various conditions, with four histamine receptor subtypes (H1-H4) identified to date [124]. As a mediator of TRPV1, histamine activates the Phospholipase A2/Lipoxygenase (PLA2/LO) pathway, stimulating sensory neurons to release neurogenic inflammatory mediators and neuropeptides, which are the direct cause of chronic itching [125]. However, sometimes antihistamines cannot completely alleviate itching, indicating that the sensation of itch involves pathways independent of histamine. Recent studies have shown that many pruritogens activate various histamine-independent itch pathways. They activate their specific receptors or ion channels without the involvement of histamine receptors [126].

The overexpression of Th2 cytokines (such as IL-4, IL-13, and IL-31) also plays a role in the pathogenesis of itching [127]. IL-4 and IL-13 have a common receptor: IL-4Rα. This receptor is present on DRG skin neurons, allowing IL-4 and IL-13 to cause itching through the sensitization of nerve fibers [128]. It has been reported that IL-4/IL-13-induced itching also depends on JAK1 signaling [129]. IL-31 can directly induce AD itching by activating neurons expressing TRPV1 and TRPA1 [130]. IL-33 is another Th2 cytokine involved in the pathogenesis of AD. In allergic contact dermatitis, IL-33 has been found to interact directly with the suppression of tumorigenicity 2 (ST2) receptor on DRG skin neurons, inducing mast cell degranulation, and regulating the production of itch-related cytokines by Th2 and other immune cells [131–133]. Studies have found that PAR2 can trigger strong TSLP expression in KCs through a calcium-dependent pathway [134], which then directly promotes itching by activating skin sensory neurons. These findings strongly demonstrate that TSLP is also a pruritogenic molecule.

#### The antipruritic effects of natural ingredients in AD treatment

Accumulating evidence highlights the therapeutic potential of natural ingredients in mitigating AD-associated pruritus through diverse mechanisms. Osthole (OST) **(17)**, a bioactive coumarin derivative, demonstrates significant anti-pruritic effects by suppressing IL-4 and IL-13 secretion in NHEK cells and 3D skin models while downregulating pruritogenic mediators. Notably, OST **(17)** upregulates TRPM8 expression and exhibits superior TRPV1 inhibitory activity compared to clobetasol propionate (CP) [135]. Recent in vivo studies reveal that OST-mediated PPARα/γ activation suppresses IL-31RA expression, suggesting PPAR modulation as a promising therapeutic target for barrier repair and itch control [136]. Substance P, a neuropeptide central to the itch-scratch cycle, promotes mast cell degranulation and histamine release [137]. AD patients exhibit increased Substance P-positive mast cells and elevated intragranular Substance P levels [138]. Therapeutic interventions demonstrate that curcumin **(18)** supplementation outperforms conventional therapies (betamethasone cream and pimecrolimus) in suppressing Substance P and alleviating pruritus [139]. Mas-related G protein-coupled receptors (Mrgpr) represent a distinct family of receptors within the GPCR superfamily. For example, MRGPRX2 is expressed by dorsal root ganglion (DRG) neurons and immune cells (such as mast cells, basophils, and EOS) and has been found to be overexpressed in itchy AD skin [140]. Mechanistic studies revealed that rosmarinic acid (RA) **(19)** binds MRGPRX2 with high affinity (confirmed by surface plasmon resonance), reducing histamine mRNA expression while upregulating MRGPRX2 in vivo [141]. *Saposhnikovia divaricata*'s primary active constituent, cimifugin **(20)**, exhibits dose-dependent competitive binding to the chloroquine receptor MrgprA3, effectively alleviating non-histaminergic itch in FITC-induced AD murine models [142].

In addition to the aforementioned single components, certain active fractions of Chinese herbal medicine have also demonstrated inhibitory effects on pruritus symptoms in AD. The anti-pruritic properties of *Artemisia argyi* volatile oil (AO) involve TRPA1 inhibition in HEK293T cells, subsequently reducing CGRP release from dorsal root ganglion neurons [143]. Fermented flaxseed oil reduces dermal Substance P immunoreactivity [144]. Furthermore, the saponin components of fermented red ginseng similarly ameliorate pruritus by regulating mast cell and eosinophil degranulation [145]. Histopathological observations reveal that AD patients exhibit abnormally dense peripheral nerve fiber projections into the epidermal layer of lesional skin, a key contributor to pruritus pathogenesis [146]. Nerve growth factor (NGF) serves as a primary mediator of this neurite outgrowth [147]. Oral administration of ImmuBalance, a fermented soybean product, directly inhibited NGF-stimulated neurite extension in neuronal cells, consequently reducing cutaneous nerve fiber density, preventing pruritus exacerbation, and suppressing scratching behavior [148].

The application of TCM extracts has also shown promising effects in improving pruritus associated with AD. Lee et al. demonstrated that *Angelica sinensis* (AS) extract markedly reduced scratching behavior in murine models, though with minimal effect on serum IgE levels, suggesting its antipruritic action primarily involves substance P modulation and mast cell regulation rather than IgE-mediated pathways [149]. TC extracts exhibited superior histamine suppression in Dermatophagoides farinae extract (DFE)-induced models compared to positive controls [80]. The ethanol extract of PUL contains bioactive components that significantly attenuated scratching behavior when administered intraperitoneally. Notably, PUL's ethyl acetate fraction inhibited TSLP-mediated calcium influx in sensory neurons through a histamine-independent mechanism, while simultaneously improving house dust mite (HDM)-induced dermatitis [126]. DCE displayed dual therapeutic benefits, not only enhancing skin barrier repair but also suppressing mast cell degranulation and reducing elevated serum levels of IgE, IL-4, TSLP, and histamine in DNCB-challenged mice, indicating its potential to ameliorate pruritus via IgE-mediated pathway inhibition [114]. The concentrated extract of *Vaccinium myrtillus* L. (Bilberry), rich in anthocyanins, effectively normalized the IL-4/IFN-γ ratio, correlating with reduced scratching behavior, though without significant impact on histamine-induced pruritus [150].

Thus, collectively, the mechanisms of natural ingredients treatment for AD-related itching symptoms mainly focus on the following aspects. For example, reducing the expression of G protein-coupled receptor (GPCR) superfamily associated with itching, such as H1R/H4R, Mrgpr, PAR, thereby blocking the binding of itching mediators to receptors and inhibiting the generation of itching sensations. In addition, it can also be achieved through inhibiting the activation of classic immune cells in the itching sensation, such as the release of histamine from mast cells and leukotriene C4 from basophils, which are inflammatory mediators that cause itching. Moreover, inhibiting the expression of cytokines and chemokines associated with atopic itching, which also act as itching mediators, thus either reducing the generation of itching sensations. Furthermore, alleviating itch symptoms can be effectively achieved by blocking the action of endogenous mediators on peripheral itching sensory neurons, reducing the skin infiltration of cutaneous nerve fibers, and inhibiting the peripheral sensitization of itching nerves. Besides, inhibiting the secretion of neurogenic inflammatory mediators and neuropeptides associated with itching in nerve cells are also possible to block the transmission of itching signals and the exacerbation of itching responses. It can also be done by inhibiting itching-related ion channels to relieve itching, such as TRPV1, TRPV4, etc. However, it has been shown that the activation of TRPA1 not only plays a role in transmitting itching signals but also contributes to the repair of the skin barrier. Therefore, any treatment that antagonizes TRPA1, while providing the benefit of relieving itching, may also have the negative impact of hindering barrier repair. As a result, researchers should consider seeking a balance between agonists and antagonists in the treatment of future atopic itching symptoms. Shown in Table 4 are the natural products that inhibit itch sensation during the development of AD-like lesions.

**Table 4:** The natural products that inhibit itch sensation during the development of AD-like lesions

Natural products	Source	Study	Dosing regimen	Positive control drug	AD model	Mast cell degranulation	scratch behavior	Itch-associated factors/protein/channels	Refs.
Osthole	*Cnidium monnieri (L) Cusson*	In vitro	0.125/0.50 mg/mL	Clobetasol propionate, 0.5 mg/mL	Histamine or LPS-Treated NHEK Cell Lines and Organotypic 3D Skin Model	—	—	IL-4, IL-13, IL-4Rα, H4R, TRPV1, TRPV4 TRPM8↓	[135]
		In vivo	Topically, 5/25/50 mg/kg, daily from day 7 to day 20	Tacrolimus, topically, 0.03%	DNCB-induced C57BL/6 mice	—	↓	IL-31, IL-31 RA histamine, IL-31↓	[136]
		In vitro	0.3/1/3 μmol/L	—	IFN-γ/TNF-α- induced HaCaT cells	—	—	IL-31, IL-31 RA↓	
Curcumin	Turmeric (powdered rhizomes of *Curcuma longa L.*)	Human research	Orally,1 g/day, daily for 4 weeks	—	Sulphur mustard-induced chronic pruritic in the human body	—	—	Substance-P↓	[139]
Rosmarinic acid	Rosmarinus officinalis L	In vivo	Subcutaneously, 1.0 mg/kg, daily from day 12 to day 14	Loratadine, subcutaneously, 1.5 mg/kg	SADBE-induced Balb/c mice	↓	↓	Histamine, IL-13, MRGPRX2↓	[141]
Cimifugin	*Saposhnikovia divaricata* (Turcz.) Schischk	In vivo	Intragastrically,25/50 mg/kg, daily from day 2 to day 7	—	FITC-Induced BALB/c mice	—	↓	—	[142]
		In vivo		—	CQ-induced BALB/c mice	—	↓	—	
*Artemisia argyi* volatile oil	*Artemisia argy*i Levl.et Vant leaves	In vivo	Subcutaneously, 30%	HC030031, subcutaneously	Chloroquine-evoked acute itch model in C57BL/6 mice	—	↓	—	[143]
		In vivo	Topically, 15/30/60%, 100 μL, daily from day 12 to day 15	Hydrocortisone butyrateointment, 100 μL	SADBE-induced chronic itch model in C57BL/6 mice	↓	↓	PGP9.5-positive neuronal fibers, CGRP, Ca + ↓	
Fermented Flax Seed Oil	Flax seeds	In vivo	Orally, 0.2 g/kg, daily for 3 weeks	—	DFE-induced NC/Nga mice	↓	—	Substance-P, Fc-epsilon Receptor, IL-4↓	[144]
*Terminalia chebula Retz.* extract		In vivo	Orally, 30/100/300 mg/kg, daily from day 9 to day 22	DXM, orally, 3 mg/kg	DFE-induced NC/Nga mice	↓	—	Histamine↓	[80]
Dictamni Cortex extract		In vitro	50/100/200 µg/ml	Ketotifen fumarate, 30 μM	Anti-dinitrophenyl IgE-sensitized RBL-2H3 cells	—	—	β-hexosaminidase, Ca + , IL-4↓	[114]
		In vivo	Orally, 0.6/1.2/2.4 g/kg, daily from day 14 to day 29	DXM, orally, 2.5 mg/kg	DNCB-induced BALB/c mice	↓	—	Histamine, IL-4↓	
*Pyrus ussuriensis Maxim.* Leaves extract		In vivo	Intraperitoneally, 2 g/kg, day 1, 4, 11, and 18	—	DNCB-induced ICR mice	—	↓	—	[126]
		In vivo	Intraperitoneally, 2 g/kg, daily for 3 weeks	—	HDM-induced NC/Nga mice	—	↓	TSLP↓	
ImmuBalance™		In vivo	Orally, 1.0%, 6–9 g, daily for 2 weeks	FK506 ointment, topically, 0.1%, 100 mg	NC/Tnd mice with moderate dermatitis	—	↓	PGP9.5-positive neuronal fibers↓	[148]
*Angelica sinensis* extract		In vivo	Topically, 20 mg/mL, 100 μL, daily for 11 days	DEX, topically, 10 μM, 100 μL	DNCB-induced BALB/c Mice	↓	↓	Substance-P, IL-4↓	[149]
Bilberry extract (Bilberon-25)		In vivo	Orally, 400/2000 mg/kg, daily from day 7 to day 17	DXM, orally, 1.5 mg/kg, every other day from day 0 to day 17	TNCB-induced BALB/c mice	—	↓	IL-4↓	[150]

—, Not Applicable or Not Determined

### Other mechanisms of action

#### Antimicrobial

The bacterial microbiota on the skin surface has always played a dual role, functioning both beneficially and detrimentally [151]. For instance, Staphylococcus epidermidis, a primary resident on healthy human skin, can inhibit inflammation after skin injury, promote wound healing, regulate skin immune processes, maintain immune tolerance to commensal bacteria, and enhance innate immune defense by inducing the expression of AMPs [152–154]. However, microbial dysbiosis appears to be a precipitating factor for certain skin diseases. Studies have shown a strong association between microbial imbalance [155] and the clinical phenotype of AD, with S. aureus being a factor that exacerbates the disease. It is well known that there is an increased colonization of Staphylococcus aureus on the skin surface of patients with AD [156], accompanied by a loss of skin bacterial diversity [157]. Moreover, recent mechanistic studies indicate that S. aureus can drive the development of AD-like lesions. When the lipids in the skin's permeability barrier are depleted to levels consistent with AD conditions, S. aureus can be internalized by KCs, translocate through tissue by transcellular passage into the SC tissue [158], or penetrate the epidermis through proteolytic mechanisms, disrupting corneocytes' ceramides or directly affecting NMFs [159, 160] and hyaluronic acid (HA) [161] to impact skin hydration; it can also reduce the mechanical integrity of the SC, leading to increased fragility of epidermal tissue, tissue rupture, and exudative lesions during AD flare-ups [162]; it may also induce B cell expansion, initiate KCs production of pro-inflammatory cytokines and stimulate mast cell degranulation, thereby leading to a Th2 skew [163, 164]; S. aureus also disrupts the proteolytic balance in the skin by inducing multiple metalloproteinases in dermal fibroblasts [165]. In summary, the failure of antimicrobial defenses or the physical skin barrier on the epidermis can enhance S. aureus's entry into the dermis, alter cytokine and AMP responses, and importantly, because this immune dysregulation further disrupts skin barrier function, it can further alter the surface microbiome and perpetuate the disease by exacerbating the barrier disruption process. Therefore, antimicrobial therapy is also an important means of treating AD.

Although some studies have shown that natural components can modulate the skin microbiota, research specifically using this mechanism to treat AD is relatively limited. First, reducing the virulence of Staphylococcus aureus represents a direct approach to modulating the skin microbiota. The study by Nakagawa et al. demonstrated that natural rosemary extracts containing carnosic acid **(21)** and carnosol **(22)** can inhibit the Agr quorum sensing system of S. aureus and suppress the virulence of S. aureus in both luciferase reporter strains and wild-type strains isolated from AD patients [166]. Similarly, the aforementioned POG **(10)**, in addition to inhibiting Th2 cell differentiation, has been shown in vitro to be a promising anti-virulence agent that attenuates S. aureus pathogenicity by targeting S. aureus caseinolytic protease P [72, 167]. Furthermore, disrupting the bacterial membrane is an effective strategy to kill S. aureus. α-mangostin **(23)**, another major extract from *Garcinia mangostana*, has been shown to prevent S. aureus infection by disrupting bacterial membranes and allowing the leakage of intracellular contents [168]. Similarly, an evaluation of the antibacterial efficacy of ethanolic extracts of Sacha inchi (*Plukenetia volubilis* L.) seed shell against S. aureus, clinical isolates, and methicillin-resistant S. aureus (MRSA) revealed that the extract exerts significant efficacy, demonstrating notable potential in reducing biofilm formation and membrane disruption properties. Several other studies have indicated that natural ingredients from Chinese herbs directly inhibit bacterial proliferation [169]. For example, antibiotic components such as hexadecanoic acid in the methanol extract of *Plantago asiatica* L. can inhibit the proliferation of harmful bacteria, including S. aureus [170]. Berberine (BBR) **(24)** is an alkaloid that attenuates the production of S. aureus toxins. Studies have shown that it inhibits bacterial growth and inflammatory responses induced by S. aureus isolated from eczema patients [171]. Moreover, in a recent study on berberine-loaded hydrogel (BHG) for the treatment of AD, in vitro antibacterial tests also showed that BHG inhibits the proliferation of S. aureus and Escherichia coli [172]. Jiawei Zicao Plaster is a traditional Chinese medicine clinically used for AD treatment. Its application to the dorsal skin of AD mice combined with 16S rRNA sequencing to characterize the skin microbiota composition revealed that JZP treatment increased the abundance of Chryseobacterium and decreased the abundance of S. aureus, which may serve as one of the mechanisms explaining its therapeutic efficacy in AD [173].

#### Anti-oxidative stress

Additionally, studies have demonstrated that elevated levels of oxidative stress (OS) are associated with the progression of chronic inflammatory skin diseases, such as AD and psoriasis [174–177]. Reactive oxygen species (ROS), including hydrogen peroxide (H_2_O_2_), superoxide anion (O^2−^), and hydroxyl radicals (OH·), are byproducts of cellular metabolism. The persistence of Th2 inflammation and the disruption of the skin barrier lead to chronic inflammation and excessive production of ROS. In addition to this mechanism, the increase in ROS may also depend on other extrinsic factors, such as solar radiation, pollution, psychological stress, and infections. Normal levels of ROS play a crucial role in cellular signaling, but over time, the excessive accumulation of ROS ultimately leads to OS, which means that the accumulation of ROS eventually exceeds the defensive capacity of the antioxidant system (AOS) [178]. Studies on animals have shown that OS can have varying degrees of negative impact on the dermal and epidermal microenvironments. In epidermal KCs, lipid peroxidation of the membrane can cause fatal oxidative damage to DNA and proteins in tissue cells, leading to cell death, while protein and lipid oxidation in the SC leads to skin barrier dysfunction and exacerbation of AD. Specifically, ROS can affect the activation of NF-κB through three representative pathways: the regulation of Inhibitor of κB (IκB) kinase (IKK) activation and inactivation, alternative phosphorylation of IκBα, and changes in the DNA-binding characteristics of NF-κB proteins [179], leading to an increase in pro-inflammatory cytokines such as IL-1β, IL-6, and TNF-α [180]. Therefore, direct oxidative damage will be exacerbated by the activation of immune mechanisms, inducing more T cell differentiation and macrophage polarization, which is closely related to the onset and exacerbation of AD symptoms [181]. Thus, inhibiting ROS-induced OS in AD lesions may be a potential strategy for treating AD. OS markers related to AD that have been identified include the transcription factor aryl hydrocarbon receptor/aryl hydrocarbon receptor nuclear translocator, nuclear factor erythroid 2-related factor 2 (NRF2), and myeloperoxidase /paraoxonase 1 activity [182].

Research on the treatment of AD through antioxidant stress by natural ingredients have been conducted. For example, curcumin **(18)**, a potent lipophilic antioxidant, can intercept lipid radicals and convert them into phenoxyl radicals within the cell membrane. It has been proven to increase antioxidant activity and reduce lipid peroxides [183]. Thus, the action of curcumin **(18)** can be defined as ROS scavenging, which can suppress ROS by increasing total glutathione (GSH) and superoxide dismutase (SOD) levels and reducing lipid peroxidation, thereby regulating the antioxidant mechanism that neutralizes free radicals. In addition, the key pharmacological mechanism of α-mangostin **(23)** in exerting antimicrobial activity is the inhibition of inflammatory and ROS oxidative enzyme activity [184]. Study results indicate that α-mangostin **(23)** can also ameliorate cellular oxidative damage caused by lipid peroxidation, enhance the cellular AOS, and increase GSH [185]. Sinomenine **(25)**, the primary active component of the traditional Chinese herb *Sinomenium acutum* (Thunb.) Rehder & E. H. Wilson, has been shown to scavenge ROS, which helps to reduce oxidative damage to skin cells due to ROS accumulation and improves skin barrier function, thereby alleviating the symptoms of AD [186]. Furthermore, extracts of *Thunbergia laurifolia* have demonstrated an increase in the activity of the AOS and a reduction in lipid peroxides through ROS scavenging in therapeutic studies on rabbit skin [185].

#### Regulation of the gut microbiota

In recent years, a growing body of research evidence has indicated that the occurrence and exacerbation of AD are not only related to skin microbiota but also to gut microbiota [187]. It is currently known that in patients with AD, the types of gut microbiota change, with a significant depletion of Bacteroides and an overexpression of Escherichia coli and Clostridium difficile [188, 189]. It is known that the gut microbiota can affect the skin through immune, metabolic, and neuroendocrine pathways [190]. The gut microbiota primarily interacts with the host through structural components and metabolic outputs, and these interactions are vital for triggering immune responses, thereby shaping innate and adaptive immunity [191]. The metabolites of gut commensal bacteria, such as Bifidobacterium, Lactobacillus, Clostridium, Bacteroides, and Streptococcus, play a major role in the proliferation of B cells and the differentiation of naive T cells into other types of Th cells and regulatory T cells (Tregs). These cells control inflammation by preventing excessive differentiation of naive T cells, downregulating cell activity, and regulating IgE production [192, 193]. The gut microbiome can also regulate cytokines and signaling pathways by breaking down metabolites derived from dietary fiber, especially short-chain fatty acids (SCFAs), affecting the activity of immune cells, thereby promoting immune system balance. It can also improve mitochondrial metabolism of damaged epidermal KCs by regulating the production of key structural components, enhancing skin barrier integrity, and thus limiting disease occurrence [194], playing an important role in regulating the pathophysiology of AD. Oral SCFAs have been shown to alleviate AD progression through their antiallergic and anti-inflammatory properties [195]. For example, studies have shown that linoleic acid and 10-hydroxy-cis-12-octadecenoic acid can alleviate AD symptoms and have a positive impact on the composition of the gut microbiome [196].

Once the gut microbiota is dysregulated, it can activate a pro-inflammatory state by altering the metabolic environment and triggering specific pattern recognition receptors (PRRs) on epithelial cells, leading to the disruption of TJs between epithelial cells. This results in increased intestinal epithelial permeability, triggering T cell activation, breaking immune suppressive cytokines and T cell-mediated tolerance, increasing the release of pro-inflammatory cytokines, exacerbating chronic inflammation, and allowing certain metabolites, toxins, and even bacteria to enter the systemic circulation [197]. When these microbes and their metabolites enter the circulation, they can promote or maintain a pro-inflammatory state in the host organism [198].

In summary, there is a complex interplay between the gut microbiota and the equilibrium of skin health. The immune system orchestrates the balance between the gut and skin through the gut-skin axis, enhancing the protective mechanisms of both barriers [199, 200]. Pathogenic interactions and inflammatory signal transduction may also impair barrier integrity, provoke immune responses, and disrupt skin immune homeostasis [201, 202]. Research on the "gut-skin" axis has unveiled new insights into the pathogenesis of AD and has paved the way for novel therapeutic interventions. Li et al. found that oral administration of ginsenoside F2 **(26)** altered the gut microbiota structure in AD mice, enriching the microbial communities that produce SCFAs, and significantly increased the levels of propionic acid (Pa) in the feces and serum of AD mice. This increase was positively correlated with the significant enrichment of Parabacteroides goldsteinii and Lactobacillus plantarum in the gut. Pa suppressed inflammatory responses in the gut and skin of AD mice through the GPR43/NF-κB pathway, thereby improving skin AD symptoms. This study is the first to reveal that ginsenoside F2 **(26)** can regulate the gut microbiota and improve AD via the Pa-gut-skin axis [203]. It has been reported that Saccharomyces, a producer of lactic acid and acetic acid, can modulate immune responses by inhibiting the expression of the TNF-α gene in macrophages [204]. Bacteria that produce large amounts of butyrate, such as Enterococcus faecalis, can alleviate the symptoms of AD [205]. Baicalin **(8)** treatment, particularly at medium and high doses, can significantly regulate the dysbiosis of DNCB-induced mice gut microbiota by restoring the abundance of Bacteroides, Faecalibacterium prausnitzii, Ruminococcus, and Lactobacillus, and reversing the abnormal growth of Bifidobacterium, Bacteroides, Mycoplasma, Parabacteroides, and Ruminococcus. Mice receiving baicalin **(8)** donor fecal microbiota had reduced skin thickness and skin EASI scores on their backs, and the release of IgE, histamine, and TNF-α in mouse serum was inhibited [70]. Emerging research has elucidated the therapeutic potential of *Senecio scandens* Buch.-Ham. polysaccharides (SSP) in modulating the gut-skin axis in atopic dermatitis. Recent investigations demonstrate that SSP administration significantly ameliorates MC903-induced dysbiosis of the intestinal microbiota, particularly enhancing α-diversity in the small intestinal microbial community. This microbiota restoration correlates with reestablished intestinal homeostasis and subsequent attenuation of AD pathogenesis [206]. Quercetin **(12)**, kaempferol **(27)**, luteolin **(5)**, and isorhamnetin **(13)** are the anti-inflammatory active ingredients of the compound Shen Chan decoction (SCD). Their administration can also improve AD symptoms by increasing the relative proportions of Lactobacillaceae and Mucoraceae at the family and genus levels, while reducing the abundance of Streptococcus and Ruminococcus, affecting the Firmicutes/Bacteroidetes ratio at the phylum level [207]. Furthermore, the Qingre-Qushi recipe (QRQS) has been shown to significantly modulate the diversity of the gut microbiota in AD-like mice by increasing Lactobacillaceae and decreasing Bacteroides. Notably, UPLC-HRMS analysis identified liquiritin **(28)** as a primary active component in QRQS, known for its potent anti-inflammatory properties. Oxymatrine **(29)** and sophoridine **(30),** key active components of this TCM, also exhibit anti-inflammatory effects. Lactobacillaceae, a well-known probiotic, not only promotes the innate immune system but also reduces serum IgE levels, maintains the balance between Th1 and Th2 responses, and strengthens the skin, immune, and intestinal barriers [208, 209]. It has been determined that the peripheral serum of FLG-deficient mice is closely related to the gut microbiota, and compared to the ft/ft group, the QRQS group showed enrichment in ascorbate and aldarate metabolism, fatty acid metabolism and biosynthesis, and propionate metabolism, suggesting that the anti-inflammatory activation mechanism of QRQS may involve improving AD-like symptoms and reducing skin inflammation by regulating the gut microbiota [210].

## In vivo studies of natural products on AD therapy

### In *vivo* efficacy studies of representative natural compounds in AD animal models

In order to investigate the pathogenesis of AD and evaluate new therapeutic methods, animal models, especially mouse models, are widely used in the research of natural ingredients for AD treatment. AD mouse models, such as C57BL/6 and BALB/c mice, are considered ideal for AD research due to their well-defined genetic backgrounds, symptom similarity to human AD, and the reliability and stability of experimental results. These models are easy to manipulate and observe, thereby reducing the cost and complexity of research and development. They not only aid in elucidating the pathogenesis and immune pathways of AD but also serve as valuable tools for testing and evaluating novel therapeutic approaches and drugs. Animal studies on the treatment of AD with natural ingredients provide critical evidence for understanding their efficacy.

In the exploration of treatments for AD, saponins from TCMs have emerged as a focal point of research due to their unique medicinal properties. Among them, ginsenosides, as a prominent representative, have demonstrated significant anti-inflammatory and immunomodulatory effects. Animal studies have revealed that ginsenosides can alleviate AD symptoms through multiple mechanisms, including skin barrier repair, itch relief, immune system regulation, and modulation of gut microbiota to indirectly improve skin inflammation, offering novel therapeutic strategies for AD. For instance, oral administration of ginsenoside F2 **(26)** has been shown to improve AD-like skin symptoms in mice via the gut-skin axis, reducing inflammatory cell infiltration, IgE levels, and the mRNA expression of inflammatory cytokines [203]. The ginsenoside fraction of Korean red ginseng (KRGS), along with its characteristic components ginsenosides Rg3 **(31)**, Rf **(32)**, and Rh2 **(33)**, significantly reduced ear swelling induced by oxazolone in mice, as well as the mRNA expression levels of TNF-α, IL-1β, COX-2, IL-4, and IFN-γ [211]. Furthermore, KRGS and its ginsenosides Rh2 **(33)** and Rg3 **(31)** were also shown to markedly decrease clinical skin severity scores, ear thickness, mast cell infiltration, and the increased mRNA expression of TNF-α and IL-4 in a TNCB-induced AD mouse model, although they did not reduce the elevated IFN-γ mRNA expression or serum total IgE levels [212]. These findings position ginsenosides as potential candidates for AD treatment. Saikosaponin A (SSA) **(34)** and tea saponin (TS), both triterpenoid saponins, have been shown to effectively restore skin damage and normalize the skin barrier in DNCB-induced BALB/c mice. SSA **(34)** significantly reduced skin thickening, immune cell infiltration, and the expression of FOS-related antigen 1 (FRA1), Jun proto-oncogene (c-Jun), and p-ERK1/2, while increasing FLG levels [213]. TS also notably decreased scratching behavior in mice, demonstrating anti-pruritic effects, and inhibited the production of inflammatory cytokines in serum and skin tissues [214].

The polysaccharides of Chinese medicine have been proved to have excellent therapeutic effects on atopic dermatitis. The active polysaccharide WLJP-025p from *Lonicera japonica* has been shown to inhibit DNCB-induced Th17 differentiation and significantly reduce serum IL-17 levels in a dose-dependent manner [215]. Studies have demonstrated that local application of *Dendrobium officinale* Kimura et Migo polysaccharide (DOP) can significantly increase FLG expression. Importantly, after DOP intervention, a significant reduction in stratum corneum thickness, reorganization of keratinocyte arrangement, and restoration of stratum corneum protein composition were observed [216]. Pine pollen polysaccharide (PPPS), a key bioactive constituent of pine pollen with therapeutic potential for atopic dermatitis (AD), exhibits a unique sponge-like architecture characterized by a three-dimensional interconnected porous network. This structure may contribute to enhanced moisturizing capacity and favorable biocompatibility. In vivo studies further revealed that PPPS administration ameliorated DNCB-induced skin damage [217]. Notably, as alluded to earlier, certain polysaccharide components might mediate anti-AD effects via the gut–skin axis—a mechanism that merits further scientific investigation.

Phenolic compounds in TCMs have garnered increasing attention in AD treatment due to their multi-target mechanisms. Among them, flavonoids have been extensively studied. Animal experiments have shown that baicalin **(8)** can improve skin barrier function by regulating the Th1/Th2 balance, enhancing the expression of barrier proteins such as FLG, IVL, and LOR, and restoring the abundance of intestinal probiotics in AD mice. It also suppresses inflammation in AD mouse models by inhibiting the activation of NF-κB and JAK/STAT pathways, thereby alleviating AD symptoms through multiple signaling pathways [70]. Chrysin **(35)**, a natural flavonoid derived from propolis, inhibits mast cell-mediated allergic reactions. Studies have demonstrated that chrysin **(35)** alleviates symptoms in DNCB/DFE-induced AD mouse models, reduces serum IgE, IgG2ɑ, and histamine levels, and suppresses Th1/Th2/Th17/Th22-related inflammatory responses in lymph nodes and ear tissues [218]. Further research revealed that chrysin **(35)** reduces mast cell infiltration into inflammatory sites and partially attenuates inflammation by targeting IKK, thereby blocking RANTES expression [219] and inhibiting TSLP expression through downregulation of Early Growth Response Protein 1 (EGR1) in the inflammatory microenvironment [220]. Quercetin **(12)**, a flavonoid aglycone widely present in most edible fruits and vegetables, exhibits antioxidant and anti-inflammatory properties. Animal studies have shown that oral quercetin **(12)** not only mitigates the development and histopathological changes of DFE-induced AD-like lesions in NC/Nga mice but also downregulates cytoplasmic high-mobility group box 1 (HMGB1), receptor for advanced glycation end products (RAGE), and nuclear levels of p-NF-κB, p-ERK1/2, COX-2, TNF-α, IL-1β, IL-2Rα, IFN-γ, and IL-4, while upregulating NRF2 levels [221]. In MC903-induced AD mouse models, quercetin **(12)** similarly reduced ear thickness, mast cell infiltration, and the expression of cytokines [222]. Curcumin **(18)** and Bisdemethoxycurcumin (BDMC) **(36)**, derived from the rhizome of *C. longa*, have been shown to lower levels of various inflammatory mediators and modulate skin inflammation, lesions, and associated atopic march diseases [223, 224]. BDMC **(36)**, more stable and with better water solubility and permeability than curcumin **(18)**, inhibits the mRNA expression of chemokines and inflammatory cytokines, as well as the activation of MAPK and NF-κB signaling pathways, thereby alleviating AD inflammation [225]. Resveratrol **(37)**, a polyphenol abundant in red grape skins, exhibits potent anti-inflammatory and anti-aging effects. Animal studies have demonstrated that resveratrol **(37)** inhibits the HMGB1-RAGE, PI3K, ERK1/2, and NF-κB pathways, suggesting HMGB1 as a potential therapeutic target for anti-inflammatory treatment [226]. Additionally, resveratrol **(37)** significantly improved skin lesions in DNCB-induced mice, reduced chemokine levels, downregulated the expression of the pro-inflammatory cytokine KLK7, and upregulated the expression of several cytokines such as FLG and Transglutaminase (TG) [227]. Chlorogenic acid (CGA) **(38)**, a bioactive polyphenol abundant in various dietary sources and medicinal plants, has been demonstrated to exhibit potent anti-inflammatory properties through topical application. Mechanistic studies reveal its ability to suppress the Akt1/NF-κB signaling pathway and downregulate histamine expression. However, despite these established anti-inflammatory effects, the potential antipruritic efficacy of CGA **(38)** remains unexplored [228].

Alkaloids, with their unique chemical properties and potent bioactivity, have attracted considerable attention in AD research as potential therapeutic targets. Dictamnine **(39)**, derived from *D. dasycarpus*, a TCM widely used in dermatological treatments, has been shown to inhibit chronic itch and inflammatory symptoms induced by DNFB in animal models, reducing mast cell infiltration, epidermal thickening, and the density of MrgprA3 + fibers in lesioned skin [229]. Further studies revealed that dictamnine **(39)** reduces macrophage infiltration, inhibits M1 macrophage polarization, upregulates microtubule-associated protein light chain 3 expression, and promotes macrophage autophagy at inflammatory sites, thereby suppressing DNFB-induced skin inflammation [230]. Pseudoephedrine **(40)**, an alkaloid from *Ephedra sinica* Stapf and *Ephedra intermedia* Schrenk & C.A.Mey., has been found to improve DNCB/DFE-induced AD-like dermatitis, skin hydration, and scratching behavior, while inhibiting serum TNF-α and IgE levels and reducing the expression of numerous cytokines and chemokines such as monocyte chemoattractant protein (MCP)-1 and matrix metalloproteinase-9 in skin tissues [231].

Terpenoids, including monoterpenes, sesquiterpenes, and diterpenes, have become a major focus of research due to their diverse structures and significant anti-inflammatory and immunomodulatory effects. Ursolic acid (UA) **(41)**, a triterpenoid, has been shown to significantly reduce dermatitis scores and ear thickness in AD mice by modulating the Toll-like Receptor 4 (TLR4)/NF-κB and NRF2/Heme Oxygenase-1 (HO-1) signaling pathways, effectively inhibiting skin proliferation, mast cell infiltration, and the expression of various pro-inflammatory factors, thereby alleviating DNCB-induced AD-like symptoms. UA **(41)** also ameliorates OS in AD mice by regulating lipid peroxidation and increasing antioxidant enzyme activity [232]. Echinocystic acid (ECA) **(42)**, a pentacyclic triterpenoid, has been demonstrated to improve skin barrier function and immune imbalance in AD by reducing epidermal/dermal thickness, immune cell infiltration, and restoring FLG expression and skin hydration. ECA **(42)** also inhibits the expression of Th cell-derived cytokines, phosphorylation of ERK and STAT1, and NF-κB translocation in KCs, highlighting its potential as an anti-atopic and anti-allergic therapeutic agent [233]. GA **(4)** has been shown to inhibit ear swelling, reduce mast cell infiltration in lesions, and modulate immune function in AD mice, thereby suppressing inflammatory responses and maintaining skin barrier stability [66].

These studies highlight the significant therapeutic effects of Chinese herbs in animal models of AD, demonstrating their ability to reduce inflammation, improve skin barrier function, alleviate itching, and enhance quality of life. These findings provide a scientific basis for natural ingredients in AD treatment and offer directions for future clinical applications. However, while animal studies are encouraging, further human clinical trials are necessary to validate the efficacy of these TCMs.

### Regulatory effects of metabolic transformation of traditional Chinese medicine active ingredients on anti-atopic dermatitis activity

The in vivo fate and therapeutic potential of active ingredients from traditional Chinese medicine are largely determined by their metabolic transformation processes in the gastrointestinal tract, liver, and local skin tissues. Following oral or topical administration, these active ingredients undergo structural modifications such as deglycosylation, methylation, hydroxylation, and hydrogenation through intestinal microbiota-mediated biotransformation, phase I/II metabolic enzyme modifications in the liver, and local metabolism in skin tissues, generating a series of metabolites [234, 235]. These metabolic transformation processes are not merely a means of drug inactivation; rather, they may significantly influence the anti-AD efficacy and clinical translational potential of these compounds by modulating their bioavailability, skin permeability, target affinity, and selectivity [236]. This characteristic represents an important feature of natural products compared to chemically synthesized drugs.

Deglycosylation is a core metabolic transformation pathway for glycoside components in traditional Chinese medicine, directly determining the bioactivity and in vivo exposure of the parent compounds [237]. Glycosides widely present in natural herbs (such as isoflavone glycosides, chromone glycosides, and ginsenosides) are highly polar, which to some extent limits their bioavailability and tissue permeability. They require deglycosylation by gut microbiota or endogenous glycosidases to generate the corresponding aglycones before effective absorption and pharmacological activity can occur [238, 239]. As mentioned in Sect. "The antipruritic effects of natural ingredients in AD treatment", the fermented soybean product ImmuBalance exerts anti-AD effects not only through the immunomodulatory actions of probiotics but also because its active material basis is closely linked to the microbial metabolism of soy isoflavones [148]: the naturally occurring isoflavone glycosides in soybeans can be converted into more bioavailable aglycones (e.g., genistein **(43)**) via microbial deglycosylation during fermentation; genistein **(43)** can then be further metabolized to a hydroxylated metabolite, orobol **(44)**. Studies have confirmed that orobol **(44)** significantly alleviates house dust mite extract (DFE)-induced AD-like skin lesions in NC/Nga mice by suppressing the overactivation of MAPK and NF-κB inflammatory signaling pathways in keratinocytes and reducing the release of Th2-type cytokines and chemokines. Its anti-inflammatory and anti-AD activities are more potent than those of the parent compound genistein **(43)** [240]. Similarly, POG **(10)**, the major component of Saposhnikovia divaricata mentioned in Sect. "The anti-inflammatory effects of natural ingredients in AD treatment", is a glycosidic prodrug that, after deglycosylation to its aglycone cimifugin **(20)**, not only retains the ability to suppress Th2 immune responses but also acquires the capacity to bind to the chloroquine receptor MrgprA3, thereby alleviating pruritus in AD mice [142]. The anti-AD activity of ginsenosides may also depend on deglycosylation metabolism: the prototype ginsenoside Rb1 **(45)** is sequentially hydrolyzed by gut microbiota into secondary saponin metabolites such as Rg3 **(31)**, Rh2 **(33)**, and F2 **(26)**. These metabolites not only exhibit significantly improved oral bioavailability but also modulate the gut–skin axis to suppress inflammatory responses in both the skin and the intestine. As noted earlier, ginsenoside F2 **(26)** ameliorates AD-like skin lesions in mice by regulating gut microbiota composition and short-chain fatty acid metabolism.

Methylation and hydroxylation modifications are the most common phase II metabolic pathways for flavonoids and polyphenols in traditional Chinese medicine, which bidirectionally regulate anti-AD activity by modulating the in vivo stability, target binding ability, and skin permeability of compounds. As mentioned above, isorhamnetin **(13)** is a methylated flavonoid derivative of quercetin **(12)** generated via biocatalytic methylation. Compared to the parent compound quercetin **(12)**, the methylated structure of isorhamnetin **(13)** significantly improves its metabolic stability in vivo and prolongs its plasma half-life [241], while also enhancing its binding affinity for PPAR-α, thereby exhibiting superior transactivation activity [104]. The polyphenol resveratrol **(37)** undergoes hydroxylation to produce its metabolite piceatannol **(46)** [242]. Compared to the parent resveratrol **(37)**, the additional hydroxyl group in piceatannol **(46)** significantly enhances its inhibitory activity against inflammatory signaling pathways such as NF-κB and MAPK, effectively reducing the release of Th2-type cytokines in AD lesions and inhibiting mast cell degranulation. At the same time, piceatannol **(46)** effectively blocks JAK1 phosphorylation in HaCaT cells and plays an important role as a multi-target inhibitor of JAK1, Syk, and PI3K [236]. Notably, the effects of methylation and hydroxylation on skin permeability are structure-dependent: moderate methylation reduces the polarity of polyphenols and enhances their percutaneous penetration, making it easier to achieve effective therapeutic concentrations locally in the skin; excessive hydroxylation, however, increases polarity and may limit transdermal absorption, providing an important theoretical basis for the design and optimization of topical TCM preparations for AD.

Overall, the metabolic transformation of active ingredients in TCM serves as a core bridge linking their chemical structures to anti-AD efficacy and is a key determinant of their clinical translation potential. Most existing studies have focused on the anti-AD pharmacological activities of the parent compounds but have often overlooked the efficacy contributions and mechanisms of their in vivo metabolites. This is one of the core reasons why some TCM ingredients show strong activity in vitro but poor efficacy in vivo. Future research on TCM for AD should pay greater attention to the in vivo metabolic processes of active ingredients, clarify the structures, activities, and targets of metabolites, and optimize administration routes and formulation designs based on metabolic transformation patterns. Only then can the multi-target, multi-pathway advantages of TCM in treating AD be fully realized, facilitating its clinical translation and application.

### Key translational barriers in current TCM-based anti-AD research

**Fig. 3:**
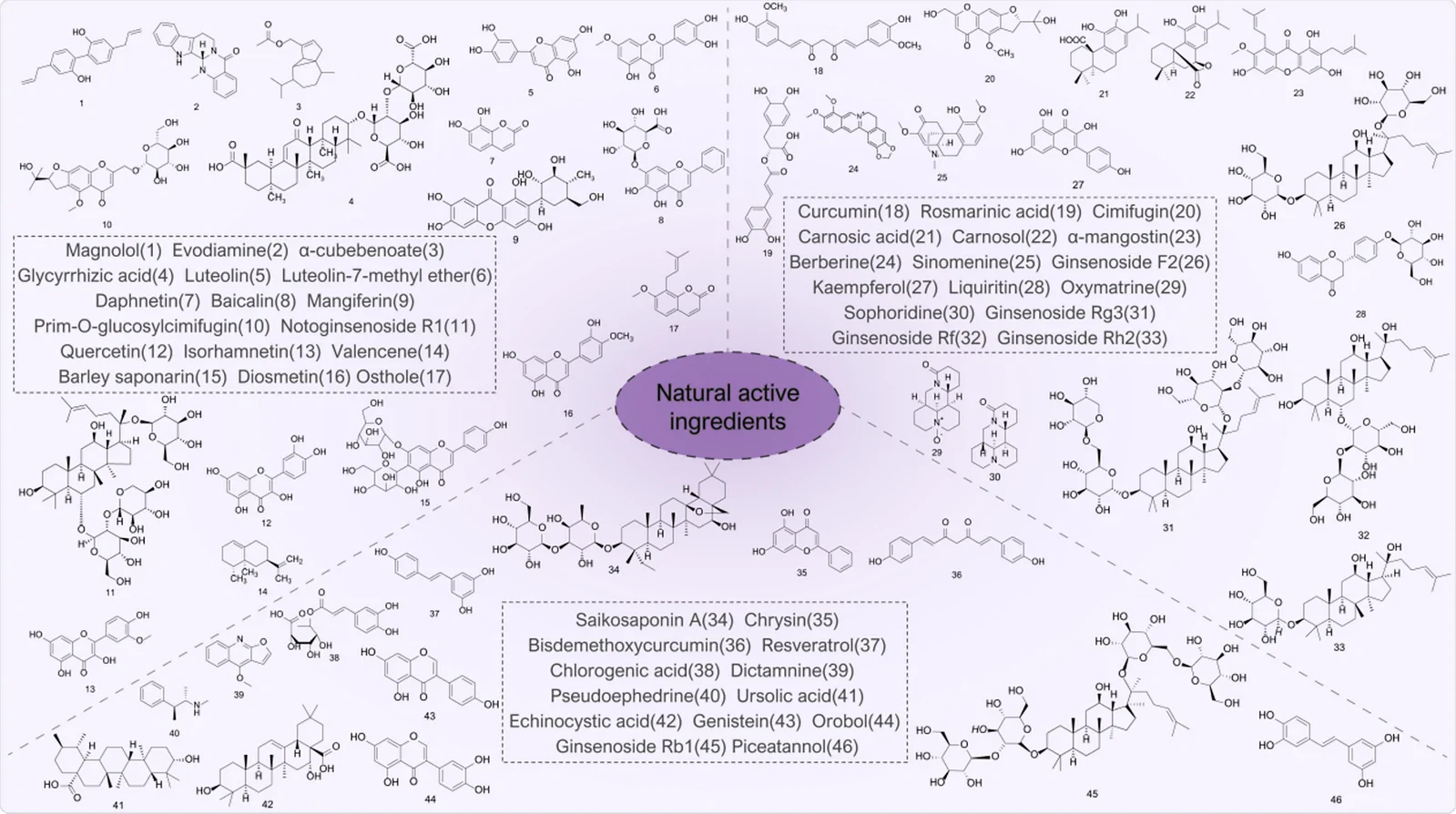
The structures of the compounds 1–46

Although TCM ingredients are often described as safe and well-tolerated, natural origin does not guarantee clinical safety. Rigorous safety evaluation is essential for clinical translation, particularly for crude extracts, compound formulations, essential oils, and complex herbal preparations. Several key safety and translational challenges must be emphasized. First, dose-dependent effects may conflict with toxicity: many TCM ingredients are safe at low doses but may cause cytotoxicity, organ damage, or pro-oxidant effects at high concentrations [243]. Therefore, dose optimization is critical to avoid adverse events. Second, certain herbal ingredients may exhibit hepatotoxicity, nephrotoxicity, or cytotoxicity when used at high doses or for prolonged periods. For example, DC is effective against AD, but the component tanshinone has been shown to easily induce acute liver injury [244]. Herbal-related organ toxicity is often underestimated in preclinical studies. Flavonoids, saponins, and polyphenols commonly suffer from poor oral bioavailability, and systemic exposure may lead to unintended off-target effects. Topical formulations may cause skin irritation, and may even induce contact dermatitis, sensitization, or pruritus. Currently, skin safety research remains insufficient, and skin irritation evaluation should be emphasized alongside pharmacodynamic validation [245–247]. Furthermore, if natural ingredients are to be developed as supplements or adjunctive therapies, whether they modulate cytochrome P450 enzymes or drug transporters and thereby interact with conventional AD treatments (e.g., corticosteroids, JAKis, antihistamines) is another important issue that requires attention [248, 249]. Most herbal preparations lack validated fingerprints, quantitative markers, or impurity detection, and contaminants (e.g., heavy metals, pesticides) further threaten their safety [250]. In summary, safety and standardization are major bottlenecks for the clinical translation of TCM-based therapies. Safety cannot be assumed due to natural origin; instead, systematic evaluations across different doses, routes of administration, and durations of use are necessary. Standardization, quality control, and targeted formulation development are indispensable for clinical application. The structures of compounds 1–46 are shown in Fig. 3.

## Summary and prospects

AD, as a prevalent chronic skin disease, presents numerous challenges in its treatment, particularly due to the side effects and efficacy limitations of current therapies. Natural ingredients, characterized by their anti-inflammatory, antioxidant, and immunomodulatory properties, offer new therapeutic opportunities. To elucidate the therapeutic potential of natural ingredients for AD, this article reviews relevant studies on the improvements of AD symptoms by natural products, and briefly introduces the research models and therapeutic mechanisms involved. Specifically, the primary mechanisms include: (1) modulating immune system function by inhibiting inflammatory cell infiltration, the production or action of pro-inflammatory cytokines, and the expression of chemokines, thereby balancing Th1/Th2/Th17/Th22 immune responses to ameliorate AD symptoms; (2) repairing the epidermal permeability barrier to improve AD skin lesions. For example, upregulating the expression of key proteins such as FLG, and improving skin hydration by enhancing lipid synthesis via the promotion of PPAR activity; and (3) inhibiting the sensation of itch, reducing scratching, and preventing further deterioration of skin lesions, primarily by reducing the expression of itch-related receptors and inhibiting the release of pruritogenic mediators from immune cells.

In addition to the principal mechanisms, natural compounds exert therapeutic effects in AD via ancillary mechanisms such as modulation of the cutaneous microbiota, mitigation of oxidative stress, and regulation of the gut microbiota. Among these, the gut–skin axis has recently garnered increasing attention, offering a novel paradigm for both mechanistic dissection and therapeutic innovation in AD. As outlined above, dysbiosis of the gut microbiome is intimately linked to immune dysregulation, skin-barrier disruption, and pruritus in AD. The central paradigm involves pharmacologic or microbiota-targeted interventions that restructure microbial composition, modulate microbial metabolites, and restore immune homeostasis—collectively ameliorating AD pathogenesis. This framework is underpinned by a robust microbiota–immune–metabolic network and has demonstrated significant efficacy in pre-clinical models and early-phase clinical trials, meriting broader investigation. Nevertheless, the response to microbiota-based therapies is modulated by dietary factors and baseline microbial signatures; therefore, establishment of predictive models enabling microbiome subtyping and precision interventions for AD warrants further exploration to advance individualized microecological therapeutics.

Overall, among the multiple pathways discussed, three mechanisms demonstrate the strongest clinical potential: modulation of the JAK–STAT/NF-κB/MAPKs signaling pathway—supported by extensive in vivo data and consistent with clinical targets; skin barrier repair through upregulation of FLG, LOR, IVL, and tight junction proteins—directly targeting the core pathological features of atopic dermatitis; and regulation of the gut–skin axis via microbial modulation and short-chain fatty acid production—representing a novel, safe, and durable long-term management strategy. Natural ingredients modulate AD pathogenesis through multi-target mechanisms, leveraging synergistic actions to address the multifactorial complexity of this disease. Continued mechanistic dissection of these pathways will accelerate the translation of natural compounds into evidence-based therapeutics that complement conventional AD management approaches. Moreover, several ingredients, such as POG **(10)**, valencene **(14)**, and TFH, have been validated in multiple animal and in vitro models and exhibit clear dose-dependent efficacy, demonstrating therapeutic potential through various in vivo and in vitro studies. Based on considerations of safety, efficacy, stability, and skin permeability, baicalin **(8)** and GA **(4)** are currently the most clinically actionable candidates, with favorable safety profiles, well-documented effects, and multiple benefits including anti-inflammatory activity, restoration of skin barrier-related proteins, and pruritus suppression. Furthermore, fermented soybean products (isoflavones/orobol) are more suitable as adjunctive oral therapies. These ingredients all hold promise for advancement into topical formulations, adjunctive oral treatments, or combination strategies.

However, it should be emphasized that most of the preclinical evidence supporting TCM ingredients for the treatment of atopic dermatitis is derived from induced animal models and in vitro cell systems. While these models are valuable for initial mechanistic exploration and efficacy screening, they cannot fully recapitulate the complexity, heterogeneity, and chronic relapsing nature of human AD. Induced animal models are mainly classified into three categories: hapten-induced models, vitamin D analog-induced models, and protein allergen-induced models. Their limitations are highly concentrated in two core aspects: deviation from human AD pathophysiology and translational shortcomings in experimental design [251]. First, the sensitizer is central to model establishment; its mechanism of action and physicochemical properties determine how well the model matches the pathogenesis of human AD, representing the most critical source of limitations [252]. Human AD typically begins with skin barrier disruption, followed by protein allergen-driven chronic mixed inflammation predominantly involving Th2 responses [253]. In contrast, haptens can penetrate intact skin and initially induce a Th1/Th17-dominated contact hypersensitivity, with only partial Th2 skewing emerging after repeated stimulation; thus, they cannot fully replicate the core vicious cycle of AD: “barrier defect—antigen invasion—immune activation—further barrier disruption” [254]. Moreover, the inherent immune bias of different mouse strains does not match the heterogeneity of human AD. For instance, inbred mouse strains exhibit natural immune polarization—C57BL/6 mice are inherently Th1-biased, BALB/c mice are Th2-biased, and NC/Nga mice exhibit spontaneous Th2 skewing [255, 256]. Human AD, however, is a mixed inflammation involving Th2, Th1, Th17, and Th22, with different disease endotypes. The polarized immune background of a single strain cannot reproduce the immune heterogeneity of human AD, and the same modeling protocol may yield completely opposite results in different strains [257]. Furthermore, translational shortcomings in treatment and dosing design are considerable. Most preclinical efficacy validations use prophylactic dosing (before or concurrent with modeling) rather than therapeutic dosing (intervention after disease onset) as applied clinically [258]. This design significantly overestimates the anti-inflammatory effects of drugs and cannot predict their actual efficacy against established chronic inflammation in humans [259]. Additionally, endpoint selection is often narrow, and validation of core biomarkers is insufficient. For example, well-established efficacy-predictive biomarkers in human AD (TARC/CCL17, IL-13, IL-31, total serum IgE) are not systematically validated in most induced models, preventing the establishment of a cross-species pharmacodynamic biomarker panel [260]. Beyond induced animal models, the core limitations of two-dimensional monolayer in vitro cell models focus on insufficient physiological relevance, inability to reconstitute the skin microenvironment, and large interspecies differences. The key pathologies of AD (barrier defects, epidermal hyperplasia) are highly dependent on three-dimensional tissue architecture, and the disease process is a vicious cycle driven by multicellular interactions [261, 262]. In vitro cell models cannot simulate the multicellular crosstalk among nerves, blood vessels, immune cells, and keratinocytes in the skin, nor can they replicate the dynamic progression from acute to chronic stages. They are only suitable for early target screening and single-pathway mechanistic validation. Most compounds that are effective in vitro prove ineffective in in vivo models, meaning that in vitro models cannot replace in vivo models for preclinical efficacy validation [263]. In summary, selecting animal models that match specific endotypes of human AD (e.g., Th2-dominant vs. Th17-skewed) and clearly defining the disease stage and clinical question (prevention vs. treatment) are key to enhancing the translational value of preclinical studies. It is recommended to adopt cross-validation using multiple models and to measure human-validated therapeutic biomarkers as core endpoints to compensate for the limitations of any single model.

Although natural ingredients have demonstrated broad promise in preclinical studies of AD, several challenges still exist. First, some natural ingredients are still at the stage of in vitro exploration, such as luteolin 7-methyl ether **(6)** and certain saponins from traditional Chinese medicine; these compounds require further in vivo validation. Second, current understanding of the composition of natural products and their structure–function relationships with biological targets remains limited. Natural ingredients are primarily extracted and isolated from Chinese medicinal herbs, and the low content of active compounds could directly affect the therapeutic effect on AD. Thus, there is an urgent need to develop effective isolation techniques to enhance the availability of natural active ingredients for therapeutic applications, as well as to further elucidate their structure-target relationships. Moreover, although natural ingredients have demonstrated significant therapeutic efficacy in the prevention and treatment of AD, the toxicological study should not be neglected. Therefore, the safety of natural active components should also be taken seriously in research. Additionally, future in-depth research integrating approaches such as network pharmacology, metabolomics, and molecular docking will facilitate the prediction, inference, and subsequent experimental validation of the structure–activity relationships and potential molecular mechanisms of TCM-derived ingredients in AD treatment. These advancements further demonstrate the promising potential of natural ingredients in the treatment of AD. Only through rigorous evaluation and rational development can TCM-based therapies become valuable sources of lead compounds‌ or adjunctive treatment options for atopic dermatitis. As research technologies continue to be applied more deeply, further insights into therapeutic mechanisms will emerge.

## Data Availability

No datasets were generated or analysed during the current study.

## Abbreviations

AD: Atopic dermatitis
AKT: Protein Kinase B
AMPs: Antimicrobial peptides
AOS: Antioxidant system
AQP-3: Aquaporin 3
CCL: C–C Motif Chemokine Ligand
CCR: C–C Chemokine Receptor
CD: Cluster of differentiation
CE: Cornified envelope
CHM: Chinese herbal medicine
COX-2: Cyclooxygenase-2
CP: Clobetasol propionate
CQ: Chloroquine
CXCL: C-X-C Motif Chemokine Ligand
DCs: Dendritic cells
DXM: Dexamethasone
DFE: Dermatophagoides farinae
dLNs: Draining lymph nodes
DNCB: 2,4-Dinitrochlorobenzene
DNFB: 2,4-Dinitrofluorobenzene
DSC1: Desmocollin 1
EASI: Eczema Area and Severity Index
EGFR: Epidermal growth factor receptor
EGR1: Early Growth Response Protein 1
EOS: Eosinophil
ERK: Extracellular-signal-regulated kinase
FLG: Filaggrin
FRA1: FOS-related antigen 1
GPCR: G protein-coupled receptor
GSH: Glutathione
HA: Hyaluronic acid
HDM: House Dust Mites
HMGB1: High-mobility group box 1
HO-1: Heme Oxygenase-1
HPA: Hypothalamic–pituitary–adrenal
IκB: Inhibitor of κB
IgE: Immunoglobulin E
IKK: IκB kinase
IL: Interleukin
IVL: Involucrin
JAK: Janus kinase
KCs: Keratinocytes
KLK: Kallikrein
LCs: Langerhans cells
LOR: Loricrin
LPS: Lipopolysaccharide
MAPK: Mitogen-activated protein kinase
MCP: Monocyte chemoattractant protein
MC903: Calcipotriol
MDC/CCL22: Macrophage-derived chemokine
Mrgpr: Mas-related G protein-coupled receptors
NF-κB: Nuclear factor kappa-light-chain-enhancer of activated B cells
NGF: Nerve growth factor
NHEK: Human Epidermal Keratinocytes
NMFs: Natural moisturizing factors
NRF2: Nuclear factor erythroid 2-related factor 2
OS: Oxidative stress
Pa: Propionic acid
PAR: Protease-activated receptor
PLA2/LO: Phospholipase A2/Lipoxygenase
PPARs: Peroxisome proliferator-activated receptors
PRRs: Pattern recognition receptors
RAGE: Receptor for advanced glycation end products
RANTES/CCL5: Regulated on Activation, Normal T Cell Expressed and Secreted
ROS: Reactive oxygen species
S. aureus: Staphylococcus aureus
SC: Stratum corneum
SCFAs: Short-chain fatty acids
SOD: Superoxide dismutase
SPINK5/LEKTI: Serine Peptidase Inhibitor Kazal Type 5
STAT: Signal transducers and activators of transcription
ST2: Suppression of tumorigenicity 2
TARC/CCL17: Thymus and activation-regulated chemokine
TCM: Traditional Chinese medicine
TCI: Topical calcineurin inhibitors
TCS: Topical corticosteroids
TEWL: Transepidermal water loss
TG: Transglutaminase
Th: T helper cell
TJ: Tight junction
TLR4: Toll-like Receptor 4
TNF-α: Tumor necrosis factor-α
TRP: Transient receptor potential
TRPA: Transient receptor potential ankyrin
Tregs: Regulatory T cells
TRPV: Transient receptor potential vanilloid
TSLP: Thymic stromal lymphopoietin
UVA1: Ultraviolet A1
UVB: Ultraviolet B
